# Cross-species transcriptomics and transgenic analyses implicate major LHCII genes in microalgal responses to ammonium stress

**DOI:** 10.3389/fpls.2026.1952007

**Published:** 2026-09-24

**Authors:** Weiyue Jin, Dan Wang, Chao Yu, Yiqin Wan, Jing Yang

**Affiliations:** School of Life Sciences, Nanchang University, Nanchang, China

**Keywords:** ammonium stress, ammonium tolerance, Auxenochlorella pyrenoidosa, Chlamydomonas reinhardtii, LHCII

## Abstract

**Introduction:**

Nitrogen is an essential macronutrient for photosynthetic organisms, but excessive ammonium causes ammonium toxicity and severely restricts growth. Although ammonium stress has been widely documented, the conserved response modules shared by microalgae and higher plants remain poorly understood.

**Methods:**

We performed a cross-species transcriptomic comparison using *Auxenochlorella pyrenoidosa, Chlamydomonas reinhardtii* and rice (*Oryza sativa*) to identify conserved pathways associated with ammonium stress. To test its functional relevance, we generated overexpression lines of the major LHCII gene in *A. pyrenoidosa* and *C. reinhardtii*.

**Results:**

The photosynthesis-antenna protein pathway was detected in all three species and significantly enriched in *C. reinhardtii* and rice, and major LHCII antenna genes were consistently downregulated under ammonium stress. Under ammonium stress, overexpression of the major LHCII gene improved algal performance in both species, but the dominant beneficial traits differed: increased cell density and chlorophyll accumulation in *A. pyrenoidosa*, and increased fresh weight together with improved chlorophyll accumulation in *C. reinhardtii*. Physiological analyses further showed species-dependent effects under the tested conditions on ROS levels and PSII effective photochemical efficiency [Y(II)], suggesting that the major LHCII gene contributes to ammonium tolerance through conserved photosynthetic functions, although the downstream physiological responses differed between the two algae under the tested conditions.

**Discussion:**

These results identify a shared transcriptional response of major LHCII genes to ammonium stress and demonstrate that their overexpression improves growth-related traits in two microalgal species.

## 1 Introduction

Nitrogen is a major component of proteins, nucleic acids and other compounds in plants, and nitrate nitrogen and ammonium nitrogen are the main nitrogen forms that plants can directly absorb from the soil. Among them, ammonium salts are efficient nitrogen sources, but high concentrations of ammonium salts act as a “double-edged sword”, affecting plant growth and development, causing oxidative damage and even death (Li et al., 2020; Liu et al., 2021; Podgórska et al., 2021; Shilpha et al., 2023; Xiao et al., 2023), a phenomenon known as ammonium toxicity. Previous studies have shown that ammonium toxicity inhibits electron transport of photosystem II and damages the oxygen-evolving complex (Wang et al., 2019; Xie et al., 2025), resulting in decreased photosynthetic efficiency and chlorophyll degradation (Alencar et al., 2019). However, the evolutionarily conserved common pathways and regulatory mechanisms in response to ammonium stress in microalgae and higher plants remain unclear (Collos and Harrison, 2014; Salbitani and Carfagna, 2021).

In photosynthesis, the light-harvesting chlorophyll-protein complex (LHC) plays a key role. LHC is encoded by the *Lhcb* gene family, binds chlorophyll a/b, and is responsible for light energy capture and transfer, driving energy to the reaction center for photochemical reactions (Büchel, 2015; Liu et al., 2026). This complex is also involved in excitation energy distribution between photosystems, photosystem protection, and thylakoid structure maintenance. The expression of *Lhcb* family proteins is highly sensitive to environmental stress, but their regulatory patterns and functional significance under ammonium stress have not been systematically studied. Ammonium toxicity mainly damages the photosynthetic system, and LHC, as the core apparatus for light energy capture, is likely to play a key regulatory role under ammonium stress through its encoding *Lhcb* gene family (Hu et al., 2025).

To explore the conservation and specificity of ammonium stress responses, this study performed a cross-species transcriptomic comparison using two green algae, *Auxenochlorella pyrenoidosa* and *Chlamydomonas reinhardtii*, together with rice (*Oryza sativa*) as a higher-plant reference. *A. pyrenoidosa* is a unicellular green alga with rapid growth and reproduction, a mature culture system, and high application potential (Bagryantseva et al., 2025). *C. reinhardtii* is also a unicellular eukaryotic green alga that has long been used as a model organism in biological research (Mussgnug, 2015; Marshall, 2024) with a well-established genetic manipulation system, facilitating mechanistic analysis. The transcriptome data of higher plant rice were included to more rigorously screen for evolutionarily conserved common pathways.

To clarify whether photosynthesis-related responses represent a conserved feature of ammonium stress and to identify functionally relevant genes underlying this response, we compared transcriptomic changes in two green microalgae, *Auxenochlorella pyrenoidosa* and *Chlamydomonas reinhardtii*, together with rice as a higher-plant reference. We aimed to (1) identify conserved pathways associated with ammonium stress across species, (2) screen candidate genes within these conserved pathways, and (3) functionally test whether overexpression of the identified LHCII genes improves performance under ammonium stress in microalgae. By integrating cross-species transcriptomics with overexpression analysis, this study provides insight into both the conserved and species-dependent features under the tested conditions of ammonium stress responses in photosynthetic organisms.

## 2 Materials and methods

### 2.1 Experimental materials

*Auxenochlorella pyrenoidosa* FACHB-9 was purchased from the Freshwater Algae Culture Bank at the Institute of Hydrobiology (Wuhan, China). *Chlamydomonas reinhardtii* CC125 was maintained in our laboratory. The higher plant material was rice (*Oryza sativa* cv. ‘ZH11’), also maintained in our laboratory. The expression vector p2300-35S was stored in our laboratory. *Escherichia coli* DH5α chemically competent cells were purchased from Shanghai Weidi Biotechnology Co., Ltd (Shanghai, China). *Agrobacterium tumefaciens* LBA4404 was obtained from Biomed Biotechnology Co., Ltd (Beijing, China).

### 2.2 Culture conditions and ammonium stress

*A. pyrenoidosa* was cultured in BG11 medium, and *C. reinhardtii* was cultured in Tris-acetate-phosphate (TAP) medium. Culture conditions: temperature 27 °C, shaking at 120 rpm, light intensity 1000 Lux, photoperiod 12 h light/12 h dark. For ammonium stress treatment, *A. pyrenoidosa* was treated with 17.6 mM NH_4_Cl (BG11 medium without sodium nitrate), and *C. reinhardtii* was treated with 35 mM NH_4_Cl. For each treatment group, the initial culture volume and initial OD_680_ were strictly controlled. Three biological replicates were set, and cells in the logarithmic growth phase were collected for subsequent experiments.

### 2.3 Transcriptome sequencing and bioinformatics analysis

*A. pyrenoidosa* was cultured under the control and ammonium stress conditions described in Section 2.2. After 6 d of cultivation, cells were harvested by centrifugation at 6, 700 rpm for 10 min at 28 °C, flash-frozen in liquid nitrogen, and stored at −80 °C. Total RNA extraction, library construction, and paired-end 150 bp sequencing were performed by Novogene Co., Ltd. (Beijing, China) on the Illumina NovaSeq 6000 platform. *C. reinhardtii* was cultured under the control and ammonium stress conditions described in Section 2.2. RNA extraction, library construction, and sequencing were performed using the same protocol as described above.

The raw RNA-seq data of *A. pyrenoidosa* and *C. reinhardtii* have been deposited in the Genome Sequence Archive (GSA) of the China National Center for Bioinformation (CNCB) under accession nos. CRA045709 and CRA045685, respectively. Rice transcriptome data (ZH11, japonica) were obtained from (Fang et al., 2021), from which the control (H_2_O) and ammonium stress (4 mM NH_4_Cl) groups (three biological replicates each) were used in this study; raw reads have been deposited in the NCBI SRA under accession no. PRJNA693667 and were re-analyzed in-house.

The A. pyrenoidosa genome assembly (GCA_047663505.1; 53.1 Mb) reported by Luo et al. (2025) was obtained from the NCBI public database (https://www.ncbi.nlm.nih.gov/datasets/genome/GCA_047663505.1/) and used as the reference. Gene annotation integrated *ab initio* prediction, transcriptome evidence, and cross-species protein homology evidence. RNA-seq reads were aligned to the genome using HISAT2, and transcript assembly was performed with Trinity (v2.15.1) and StringTie v2.2.1, followed by open reading frame prediction with TransDecoder and gene structure refinement with the PASA pipeline (v2.5.3, minimap2 aligner). Cross-species protein evidence was obtained by aligning protein sequences from *Arabidopsis thaliana* (TAIR10), *Oryza sativa* (IRGSP-1.0), *Zea mays* (B73), *C. reinhardtii* (Phytozome v5.6), and *Chlorella vulgaris* to the genome using miniprot. Repetitive elements were soft-masked with RepeatModeler and RepeatMasker, and *ab initio* gene prediction was performed with AUGUSTUS. All three evidence types were integrated using EVidenceModeler, and the longest protein-coding transcript per gene was retained.

RNA-seq reads of the three species were aligned to their respective reference genomes (*A. pyrenoidosa*: GCA_047663505.1; *C. reinhardtii*: v5.0; rice: IRGSP-1.0) using HISAT2, and fragment-level counts were generated with featureCounts. Fourteen of the fifteen original samples were included in the analysis; one control replicate of *C. reinhardtii* (A1) was not used because only single-end reads were available. Differential expression analysis was performed with DESeq2 for all three species with the same thresholds (|log_2_FoldChange| > 1, padj < 0.05). Quality control of biological replicates was performed by principal component analysis and Pearson correlation analysis (**Supplementary Figure A1**). KEGG pathway information was obtained from eggNOG-mapper functional annotation, and KEGG enrichment analysis was performed on the DEGs of each species using the enricher function in clusterProfiler with hypergeometric testing. Pathways with *P* < 0.05 were considered significantly enriched, and padj are additionally reported (**Supplementary Table 1**). For cross-species comparison and visualization, all significantly enriched pathways (*P* < 0.05) of each species were used. Significantly enriched pathways shared by at least two species were identified; shared pathways related to photosynthesis or carbon fixation are shown in **Figure 1**, and the overlap of all significantly enriched pathways among the three species is shown in **Supplementary Figure A2**.

**Figure 1:**
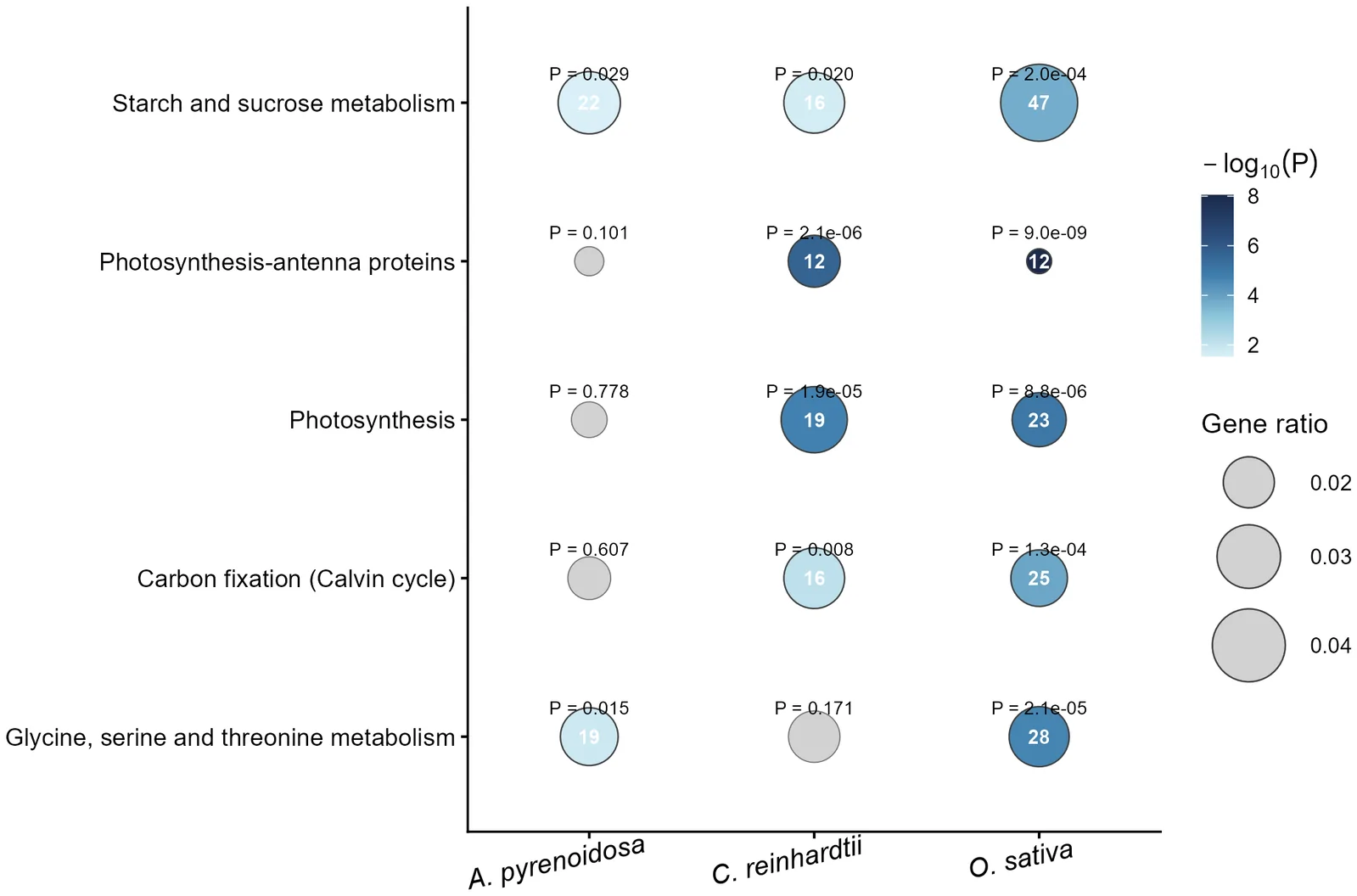
Cross-species comparison of significantly enriched KEGG pathways under ammonium stress. The five significantly enriched pathways shared by at least two species that are related to photosynthesis or carbon fixation are shown. Bubble size represents the gene ratio (DEGs in the pathway/DEGs with KEGG annotation), bubble color represents −log_10_ (*P*), and the number inside each bubble is the number of DEGs in that pathway. Grey circles mark pathway–species combinations in which the pathway was not significantly enriched. The photosynthesis-antenna protein pathway (ko00196) was significantly enriched in both *C. reinhardtii* (P = 2.06 × 10^−6^) and rice (P = 8.99 × 10^−9^) but not in *A. pyrenoidosa*.

To identify cross-species orthologous genes, the longest protein-coding transcript per gene was selected for each species. Orthologous groups were inferred using OrthoFinder v3.1.4 with DIAMOND v2.2.0 for protein sequence alignment and MCL clustering (inflation = 1.5). Orthogroups containing genes from all three species were defined as cross-species conserved. Candidate genes were identified among the members of the two focal antenna orthogroups (major LHCII and LHCA4) in the photosynthesis-antenna protein pathway, as described in the Results.

To verify gene identity across species, LHCB family protein sequences from *A. thaliana*, *O. sativa*, *C. reinhardtii*, and *A. pyrenoidosa* were aligned using MAFFT v7 (--auto algorithm). A maximum-likelihood phylogenetic tree was constructed with FastTree v2 (LG + Gamma substitution model).

### 2.4 Generation and screening of transgenic lines

Total RNA was extracted using the Karroten Total RNA Extraction Kit and reverse-transcribed into cDNA. The coding sequence of the major LHCII gene (*LHCBM3* in *C. reinhardtii*; an LHCII family member in *A. pyrenoidosa*) was amplified by PCR using the cDNA as template, and then cloned into the p2300-35S expression vector via homologous recombination. After verification, the recombinant plasmid was introduced into *Agrobacterium tumefaciens* LBA4404 by plasmid DNA transformation. Subsequently, the candidate gene was transformed into *A. pyrenoidosa* and *C. reinhardtii* using the *Agrobacterium*-mediated transformation method. To determine the appropriate selection concentration for each alga, *A. pyrenoidosa* and *C. reinhardtii* were inoculated on solid media containing different concentration gradients of G418, and the minimum effective concentration that completely inhibited wild-type growth was selected for screening positive transgenic lines. The transgenic lines were further identified by PCR and quantitative real-time PCR (qRT-PCR), and sequencing was performed to confirm positive transgenic lines. From the verified transgenic lines, ten (for *A. pyrenoidosa*) and six (for *C. reinhardtii*) independent lines were retained; three lines per species were randomly selected from these retained lines for the physiological experiments.

### 2.5 Growth and physiological index measurements

For the physiological experiments, three independent transgenic lines per species were randomly selected from the verified overexpression lines. Wild-type and corresponding transgenic lines of *A. pyrenoidosa* and *C. reinhardtii* were cultured under normal and stress conditions until the stationary phase. OD_680_ was measured daily and changes in algal solution color were recorded. After the stationary phase, the following physiological indices were measured: cell density was counted using a hemocytometer; fresh weight was determined after centrifugation of algal cells (12, 000 rpm, 10 min); chlorophyll a and b contents were determined by the methanol extraction method; reactive oxygen species (ROS) levels were detected using the Beyotime Reactive Oxygen Species Assay Kit (S0033S); superoxide dismutase (SOD) activity was measured using the Nanjing Jiancheng WST-1 assay kit (A001-3-1); chlorophyll fluorescence parameters Fv/Fm and Y(II) were measured using a chlorophyll fluorometer after 15 min of dark adaptation.

### 2.6 Data processing and graphing

All data are presented as mean ± SD of three biological replicates. Bar graphs (fresh weight, cell density, chlorophyll content, SOD, ROS, and Fv/Fm) were analyzed by Welch’s ANOVA with Dunnett’s T3 *post-hoc* test for comparisons between each transgenic line and the wild-type control. Growth curves (OD_680_) and Y(II) time-course line graphs were analyzed by two-way ANOVA (Time × Strain) followed by Dunnett’s *post-hoc* test at individual time points. Relative increase ratios (OE line value/mean of three WT biological replicates) were compared between normal and ammonium stress conditions using paired two-tailed Student’s t-test. Differences were considered significant at *P* < 0.05, highly significant at *P* < 0.01 and *P* < 0.001. All graphs were generated using GraphPad Prism 10.6.0.

## 3 Results

### 3.1 The photosynthesis-antenna protein pathway is detected in all three species under ammonium stress and major LHCII genes are identified as candidate genes

Through the comprehensive annotation pipeline, a total of 8, 278 protein-coding genes were obtained for the *A. pyrenoidosa* genome. To investigate key metabolic pathways under ammonium stress, transcriptome sequencing was performed on *A. pyrenoidosa*, and 3, 940 differentially expressed genes (DEGs) were identified (|log_2_FoldChange| > 1, padj < 0.05). KEGG enrichment analysis was conducted based on gene functional annotation (**Figure 1**), revealing that 10 pathways were significantly enriched in *A. pyrenoidosa* (P < 0.05; **Supplementary Table 1**), including ribosome, purine metabolism, glycine-serine-threonine metabolism, nicotinate and nicotinamide metabolism, drug metabolism–other enzymes, and DNA replication.

Using existing transcriptome data of *C. reinhardtii* and rice, the KEGG enrichment results of the three species were further compared (**Figure 1**). In *C. reinhardtii* and rice, 24 and 38 pathways were significantly enriched, respectively, including photosynthesis-antenna proteins, photosynthesis, and the Calvin cycle; in *A. pyrenoidosa*, 10 pathways were significantly enriched. Among the significantly enriched pathways of the three species, seven were shared by at least two species, of which five are closely related to photosynthesis or carbon fixation: starch and sucrose metabolism (ko00500), significantly enriched in all three species; photosynthesis-antenna proteins (ko00196) and photosynthesis (ko00195), significantly enriched in *C. reinhardtii* and rice; carbon fixation by the Calvin cycle (ko00710), significantly enriched in *C. reinhardtii* and rice; and glycine, serine and threonine metabolism (ko00260), significantly enriched in *A. pyrenoidosa* and rice. The remaining two shared pathways (drug metabolism–other enzymes and microbial metabolism in diverse environments) were not related to photosynthesis or carbon metabolism. Notably, the photosynthesis-antenna protein pathway was the most significantly enriched pathway in *C. reinhardtii* (*P* = 2.06 × 10^−6^) and was also strongly enriched in rice (*P* = 8.99 × 10^−9^); in *A. pyrenoidosa*, it appeared in the pathway list but did not reach the threshold of statistical significance. Given that this pathway was significantly enriched in both *C. reinhardtii* and rice, it was selected as a cross-species conserved ammonium stress-responsive pathway for subsequent analysis. The complete enrichment results and the overlap of all significantly enriched pathways among the three species are provided in **Supplementary Table 1** and **Supplementary Figure A2**.

To screen for conserved ammonium stress-responsive genes, cross-species orthologous gene analysis was performed among the three species. The protein sequence inputs for orthologous analysis were 8, 278 (*A. pyrenoidosa*, this study), 17, 741 (*C. reinhardtii*, Phytozome v5.6), and 35, 682 (rice, IRGSP-1.0). Clustering yielded a total of 9, 285 orthogroups, of which 3, 009 contained genes from all three species and were designated as conserved orthogroups, providing a basis for subsequent candidate gene screening.

A total of 42 strict DEGs were annotated to the photosynthesis-antenna protein pathway across the three species (**Supplementary Table 2**). The candidate set was defined by the study focus rather than by statistical ranking: the major LHCII family (the dominant PSII antenna) and the LHCA4 family (the second conserved antenna family identified in the cross-species screen). The complete three-species members of these two focal orthogroups comprised eight candidate orthologs forming three ortholog groups (**Table 1**; expression statistics in **Table 2**). Gene identities were verified by phylogenetic analysis (**Supplementary Figure A3**). Seven of the eight members satisfied the strict DEG criteria in their own species; the remaining one, *LHCBM3* (*Cre04.g232104*), is the ortholog-pair member defined by the cross-species ortholog comparison and is reported without being claimed as a significant DEG. *CM105676.1.547* and *CM105675.1.918* are the *A. pyrenoidosa* members of the two LHCA4 ortholog pairs (paired with *Cre10.g425900* and *Cre06.g278213*, respectively), and both carry an unambiguous *LHCA4* annotation (single K08910). The significant LHCA4-family member *CM105675.1.917* was not used as a pair representative because its dual *LHCA4*/*LHCA5* annotation (K08910+K08911) leaves its subfamily assignment ambiguous. *LHCBM3* was not a significant DEG in *C. reinhardtii* (log_2_FC = −1.26, padj = 0.171; **Table 2**) and was therefore selected as the *C. reinhardtii* representative of the major LHCII group on orthology and functional grounds rather than as a significant DEG: its orthologs were significantly downregulated in *A. pyrenoidosa* (log_2_FC = −1.49) and rice (log_2_FC = −9.74), and *LHCBM3* is one of the best-characterized LHCBM proteins of the PSII peripheral antenna (Elrad et al., 2002). Among the three ortholog groups, the major LHCII group was prioritized for functional validation. First, the dominant phenotypes of ammonium toxicity — chlorosis and chlorophyll degradation — indicate PSII damage, and major LHCII is the dominant PSII antenna protein, making it the most mechanistically relevant candidate; LHCA4 belongs to the PSI antenna and is less directly linked to these phenotypes. Second, among the members passing the significance threshold, the major LHCII group showed the strongest downregulation in rice (−9.74 vs. −8.97 for LHCA4), and in *A. pyrenoidosa* the major LHCII gene showed the strongest downregulation among the candidate orthologs (log_2_FC = −1.49 vs. −1.26 and −1.28 for the two *LHCA4* paralogs).

**Table 1:** Cross-species candidate ortholog groups in the photosynthesis-antenna protein pathway.

Orthogroup	*Auxenochlorella pyrenoidosa*		*Chlamydomonas reinhardtii*		*Oryza sativa*	
	Gene ID	Annotation	Gene ID	Annotation	Gene ID	Annotation
OG0000187	*evm.TU.CM 105675.1.840*	LHCII	*Cre04.g232104.v5.5*	*LHCBM3*	*Os03g0592500*	*LHCB2*
OG0000456	*evm.TU.CM 105676.1.547*	*LHCA4*	*Cre10.g425900.v5.5*	*LHCA4*	*Os08g0435900*	*LHCA4*
	*evm.TU.CM 105675.1.918*	*LHCA4*	*Cre06.g278213.v5.5*	*LHCA4*		

The eight candidate orthologs derive from two cross-species orthogroups (OG0000187, major LHCII; OG0000456, *LHCA4*) and form three ortholog groups: one major LHCII group and two *LHCA4* paralog pairs. Seven of the eight members satisfied the strict DEG criteria (|log_2_FC| > 1, padj < 0.05) in their own species (**Supplementary Table 2**). The remaining one — *LHCBM3* (*Cre04.g232104*) — is the ortholog-pair member defined by the cross-species ortholog comparison and is reported for cross-species comparison without being claimed as a significant DEG. In *C. reinhardtii*, *Cre10.g425900.v5.5* and *Cre06.g278213.v5.5* are both annotated as *LHCA4*, representing distinct paralogs within the same gene family.

**Table 2:** Expression of the candidate orthologs under control (A) and ammonium stress (B) conditions.

Gene ID	A1	A2	A3	B1	B2	B3	log_2_FC	padj
*Cre04.g232104.v5.5*		46458	58486	7183	27000	18544	-1.26	0.171
*Cre10.g425900.v5.5*		25903	31904	7350	10667	8979	-1.41	2.2×10^-8^
*Cre06.g278213.v5.5*		9383	16049	2025	4961	4616	-1.40	0.0443
*evm.TU.CM105675.1.840*	4155	14146	9794	7853	5885	4850	-1.49	4.7×10^-5^
*evm.TU.CM105676.1.547*	1159	5408	3380	3345	2123	2089	-1.26	6.1×10^-3^
*evm.TU.CM105675.1.918*	2667	7851	5503	4833	3822	3508	-1.28	8.3×10^-5^
*Os03g0592500*	723	535	499	0	1	1	-9.74	3.0×10^-20^
*Os08g0435900*	428	278	325	0	1	1	-8.97	3.4×10^-17^

A = control; B = ammonium stress; A1–A3 and B1–B3 are biological replicates. For *C. reinhardtii*, the A1 replicate is not shown because only single-end reads were available for this sample. log_2_FC and padj were calculated by DESeq2 with uniform thresholds across species. Seven of the eight genes satisfy the strict DEG criteria (|log_2_FC| > 1, padj < 0.05).

### 3.2 Construction of the major LHCII gene expression vector and generation of transgenic overexpression lines

The target major LHCII gene was successfully cloned from *C. reinhardtii* and *A. pyrenoidosa*, and the p2300-35S overexpression vector was constructed. The vector was verified by sequencing, and the sequence was consistent with the expected design.

Wild-type *C. reinhardtii* and *A. pyrenoidosa* were cultured on solid media containing different concentrations of antibiotics. *C. reinhardtii* failed to grow on solid medium containing ≥5 μg/mL G418; *A. pyrenoidosa* failed to grow on solid medium containing ≥25 μg/mL G418. Accordingly, the minimum effective selection concentration was determined as 5 μg/mL G418 for *C. reinhardtii* and 25 μg/mL G418 for *A. pyrenoidosa*.

qRT-PCR analysis showed that the transcript levels of the major LHCII gene were higher than those in the wild type in all verified overexpression lines of both algae, with statistically significant increases (*P* < 0.05) in three *A. pyrenoidosa* lines (OE-ApLHCII-3, OE-ApLHCII-6, and OE-ApLHCII-7) and five *C. reinhardtii* lines (OE-CrLHCBM3-1, OE-CrLHCBM3-2, OE-CrLHCBM3-3, OE-CrLHCBM3-8, and OE-CrLHCBM3-9), indicating successful construction of the overexpression lines (**Figure 2**).

**Figure 2:**
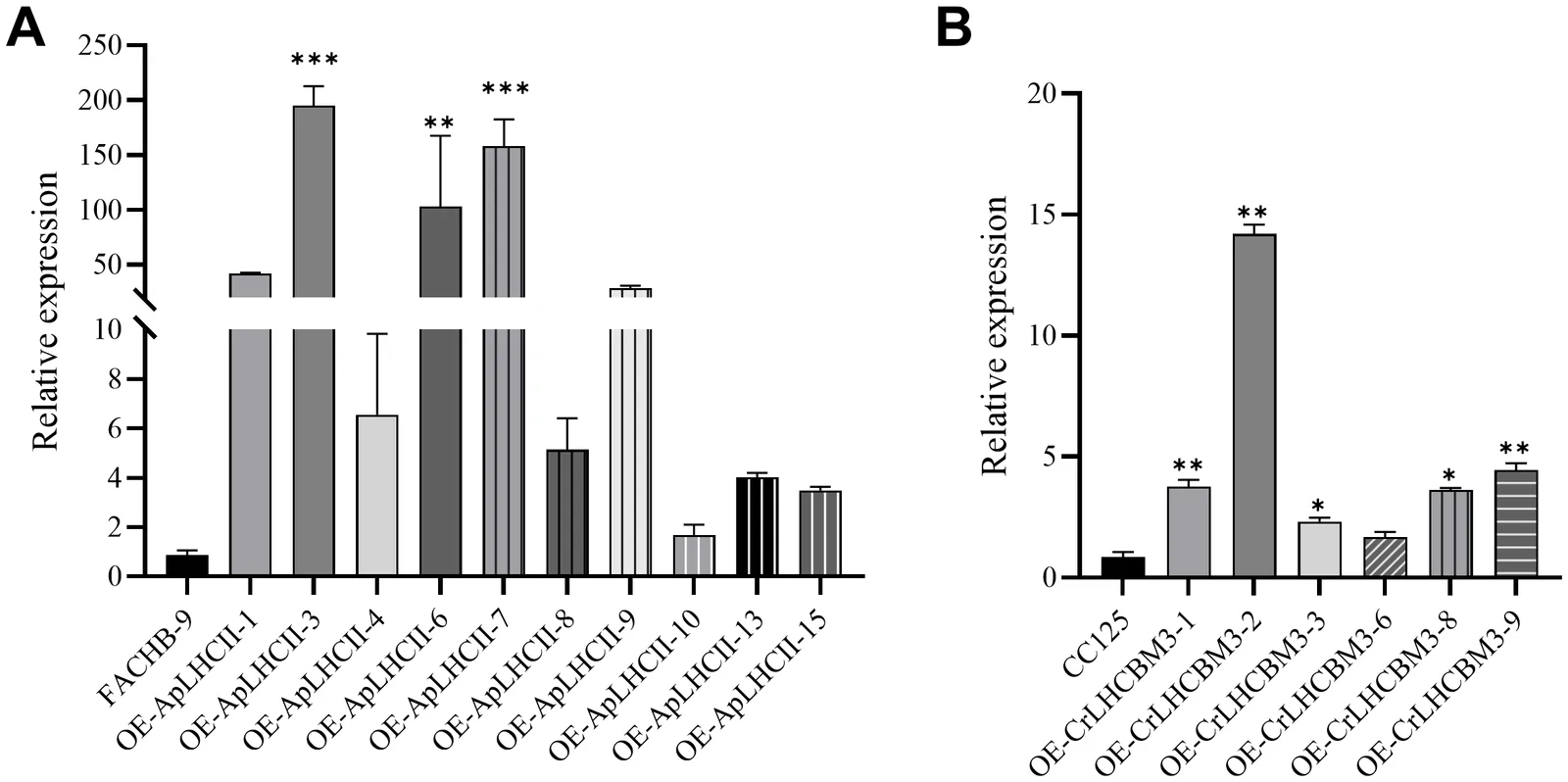
qRT-PCR validation of LHCII overexpression in both algae. **(A)** *A. pyrenoidosa* (*ApLHCII*); **(B)** *C. reinhardtii* (*CrLHCBM3*). Relative expression levels were normalized to the respective wild-type (set to 1). Data are presented as mean ± SD (*n* = 3). Differences between each transgenic line and WT were assessed by Welch’s ANOVA with Dunnett’s T3 *post-hoc* test.*P < 0.05, **P < 0.01, ***P < 0.001 vs WT.

### 3.3 Overexpression of the major LHCII gene significantly promotes growth, cell proliferation and chlorophyll accumulation in *C. reinhardtii* and *A. pyrenoidosa* under normal conditions

Under normal conditions, the algal solution color of OE lines in both algae was deeper than that of the wild-type (**Figures 3A, B**). Growth curves showed that OE lines exhibited higher OD_680_ than the wild-type (**Figures 3C, D**). Except for a few lines, the fresh weight, cell density, and chlorophyll a and b contents of OE lines in both algae were significantly or highly significantly higher than those of the wild-type (**Figures 3E–L**).

**Figure 3:**
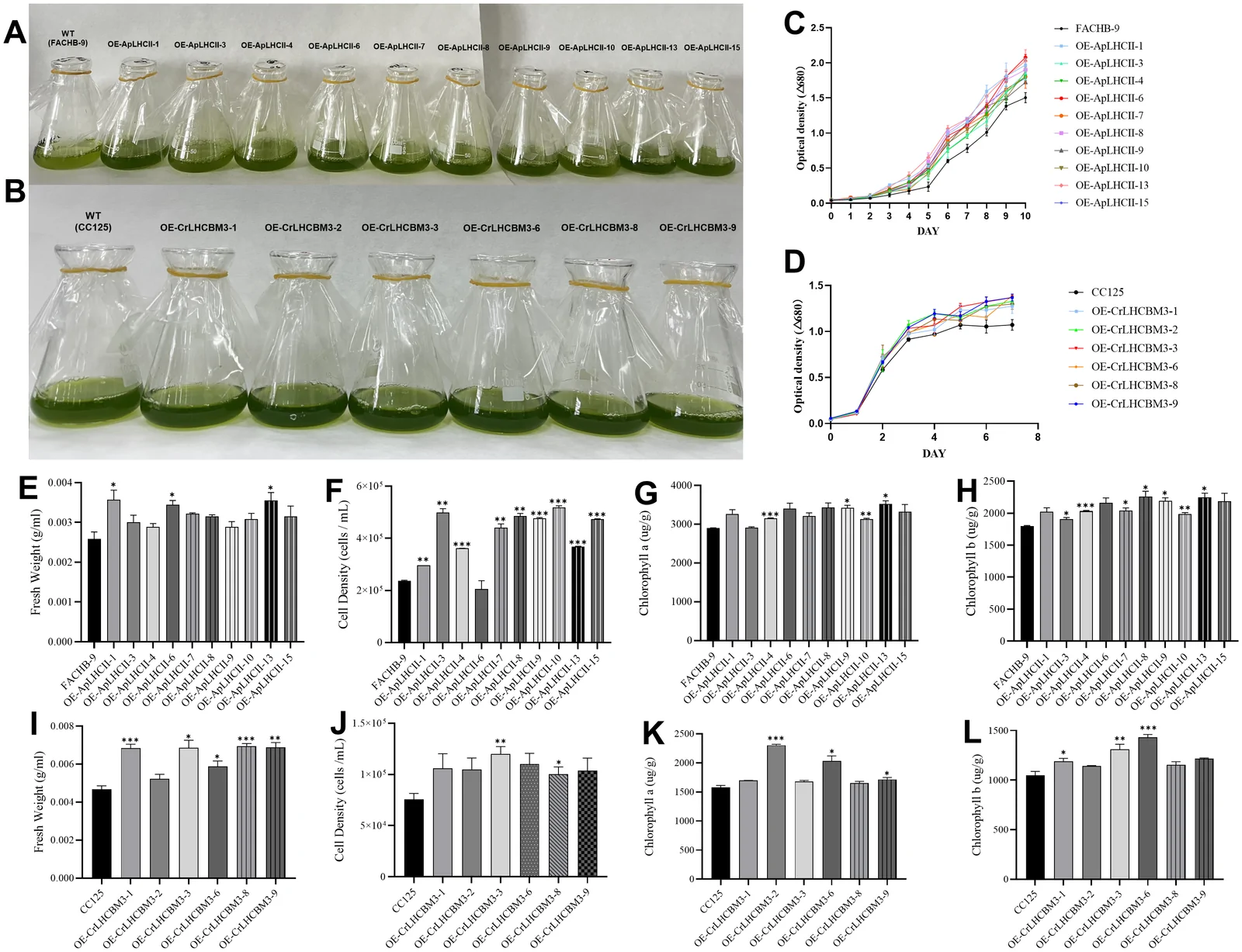
LHCII overexpression promotes growth and chlorophyll accumulation under normal conditions in *A. pyrenoidosa* and *C. reinhardtii.* **(A)** Algal color of *A. pyrenoidosa* on day 5. WT: wild type; OE-CrLHCBM3 and OE-ApLHCII: overexpression lines in *C. reinhardtii* and *A. pyrenoidosa*, respectively; **(B)** Algal color of *C. reinhardtii* on day 3; **(C)** Growth curve (OD_680_) of *A. pyrenoidosa*; **(D)** Growth curve (OD_680_) of *C. reinhardtii*; **(E)** Fresh weight of *A. pyrenoidosa*; **(F)** Cell density of *A. pyrenoidosa*; **(G)** Chlorophyll a content of *A. pyrenoidosa*; **(H)** Chlorophyll b content of *A. pyrenoidosa*; **(I)** Fresh weight of *C. reinhardtii*; **(J)** Cell density of *C. reinhardtii*; **(K)** Chlorophyll a content of *C. reinhardtii*; **(L)** Chlorophyll b content of *C. reinhardtii*. For panels **(C, D)**, data are mean ± SD (n = 3); strain and interaction effects were assessed by two-way ANOVA (Time × Strain) followed by Dunnett’s *post-hoc* test, with full per-day comparisons provided in **Supplementary Table 4** and **Supplementary Table 5**. For panels **(E–L)**, data are mean ± SD (n = 3) and were analyzed by Welch’s ANOVA with Dunnett’s T3 *post-hoc* test. *P < 0.05, **P < 0.01, ***P < 0.001 vs respective wild type. ns, not significant.

### 3.4 Overexpression of the major LHCII gene enhances ammonium tolerance in *A. pyrenoidosa* and *C. reinhardtii*

Under ammonium stress (35 mM NH_4_Cl for *C. reinhardtii*, 17.6 mM NH_4_Cl for *A. pyrenoidosa*), the algal color of OE lines in both algae was deeper than that of the wild-type, and the chlorosis rate was slower (**Figures 4A, B**). For *A. pyrenoidosa*, the wild-type showed significant growth inhibition and chlorosis on days 4–5, whereas *C. reinhardtii* showed similar phenotypes on day 7.

**Figure 4:**
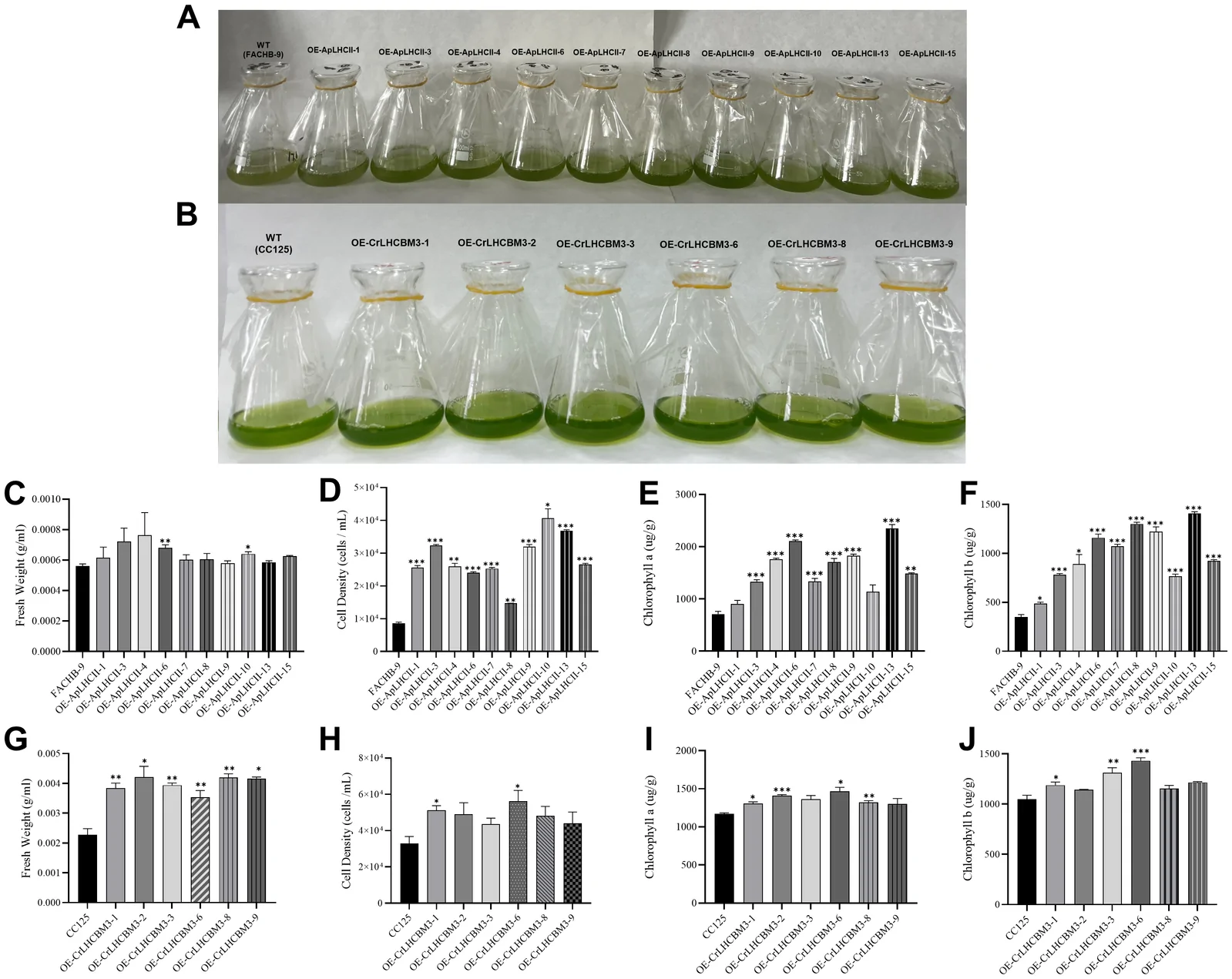
Under ammonium stress, Overexpression of the major LHCII gene delays chlorophyll degradation in *A. pyrenoidosa* and *C. reinhardtii*, increasing cell density and chlorophyll content in *A. pyrenoidosa* and fresh weight, cell density and chlorophyll content in *C. reinhardtii*. **(A)** Algal color of *A. pyrenoidosa* (WT vs OE lines); **(B)** Algal color of *C. reinhardtii*; **(C)** Fresh weight of *A. pyrenoidosa*; **(D)** Cell density of *A. pyrenoidosa*; **(E)** Chlorophyll a content of *A. pyrenoidosa*; **(F)** Chlorophyll b content of *A. pyrenoidosa*; **(G)** Fresh weight of *C. reinhardtii*; **(H)** Cell density of *C. reinhardtii*; **(I)** Chlorophyll a content of *C. reinhardtii*; **(J)** Chlorophyll b content of *C. reinhardtii*. Data are mean ± SD (n = 3). Differences between each transgenic line and the wild type were assessed by Welch’s ANOVA with Dunnett’s T3 *post-hoc* test. *P < 0.05, **P < 0.01, ***P < 0.001 vs respective wild type; ns, not significant.

Physiological index measurements at the stationary phase showed that in *A. pyrenoidosa* (**Figures 4C–F**), OE lines exhibited significantly or highly significantly higher cell density and chlorophyll a/b contents than the wild-type, but fresh weight was not significantly different (except for a few lines). In *C. reinhardtii* (**Figures 4G–J**), all OE lines showed highly significantly higher fresh weight and chlorophyll content than the wild-type, and cell density was also significantly or highly significantly increased (except for a few lines).These results support a positive contribution of overexpression of the major LHCII gene to ammonium tolerance in both algae, although the dominant beneficial traits differed: cell density and chlorophyll content in *A. pyrenoidosa*, versus fresh weight and chlorophyll content in *C. reinhardtii*.

Relative increase ratio analysis (**Figure 5**) further showed that, compared with normal conditions, the relative increase ratios of cell density and chlorophyll content in *A. pyrenoidosa* OE lines under ammonium stress were significantly higher, while the relative increase ratio of fresh weight was not significantly different. For *C. reinhardtii*, the relative increase ratio of fresh weight under ammonium stress was significantly higher, while those of cell density and chlorophyll a/b were not significantly different.

**Figure 5:**
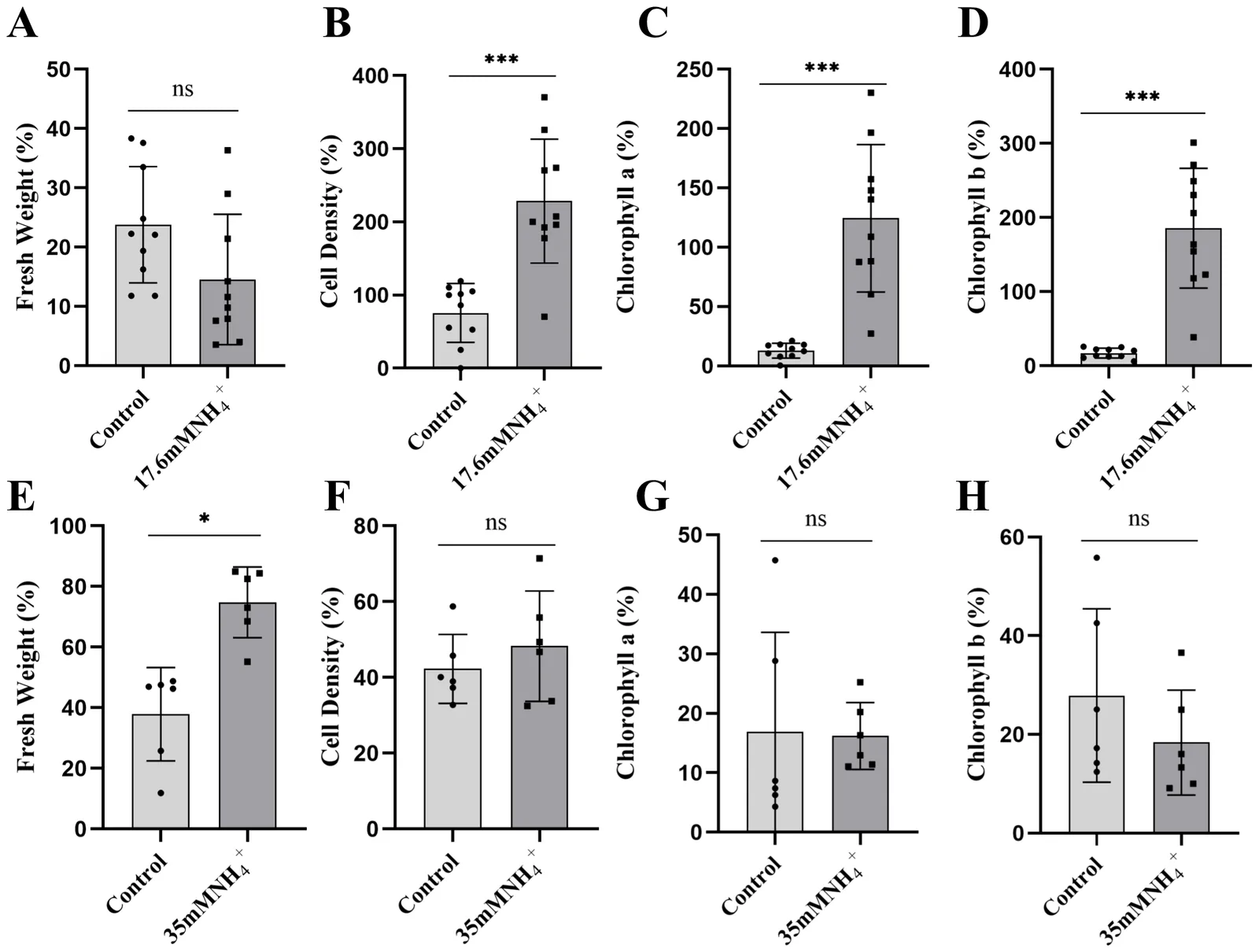
Relative increase ratios of LHCII overexpression on cell density and chlorophyll in *A. pyrenoidosa* and on fresh weight in *C. reinhardtii* are greater under ammonium stress than under normal conditions. **(A)** Relative increase ratio of fresh weight in *A. pyrenoidosa*; **(B)** Relative increase ratio of cell density in *A. pyrenoidosa*; **(C)** Relative increase ratio of chlorophyll a in *A. pyrenoidosa*; **(D)** Relative increase ratio of chlorophyll b in *A. pyrenoidosa*; **(E)** Relative increase ratio of fresh weight in *C. reinhardtii*; **(F)** Relative increase ratio of cell density in *C. reinhardtii*; **(G)** Relative increase ratio of chlorophyll a in *C. reinhardtii*; **(H)** Relative increase ratio of chlorophyll b in *C. reinhardtii*. Data are mean ± SD, n = 10 (*A. pyrenoidosa*) or n = 6 (*C. reinhardtii*) independent transgenic lines. *P* values were determined by paired two-tailed Student’s t-test comparing the same line under normal vs. ammonium stress conditions. *P < 0.05, **P < 0.01, ***P < 0.001; ns, not significant.

### 3.5 Overexpression of the major LHCII gene is associated with species-dependent changes in ROS homeostasis and PSII photochemical performance

The effects of overexpression of the major LHCII gene on ROS homeostasis and PSII photochemical performance differed between the two algae under the tested conditions. Measurement of reactive oxygen species (ROS) levels at the stationary phase (**Figures 6A–D**) showed that under normal conditions, no significant difference in ROS content was observed between wild type and OE lines in *A. pyrenoidosa*. Under ammonium stress, ROS levels in all lines of *A. pyrenoidosa* increased compared with normal conditions, but except for a few lines, ROS content in OE lines was significantly lower than that in the wild-type. In *C. reinhardtii*, ROS levels in OE lines were lower than those in the wild-type under both normal and ammonium stress conditions, but the differences were not significant.

**Figure 6:**
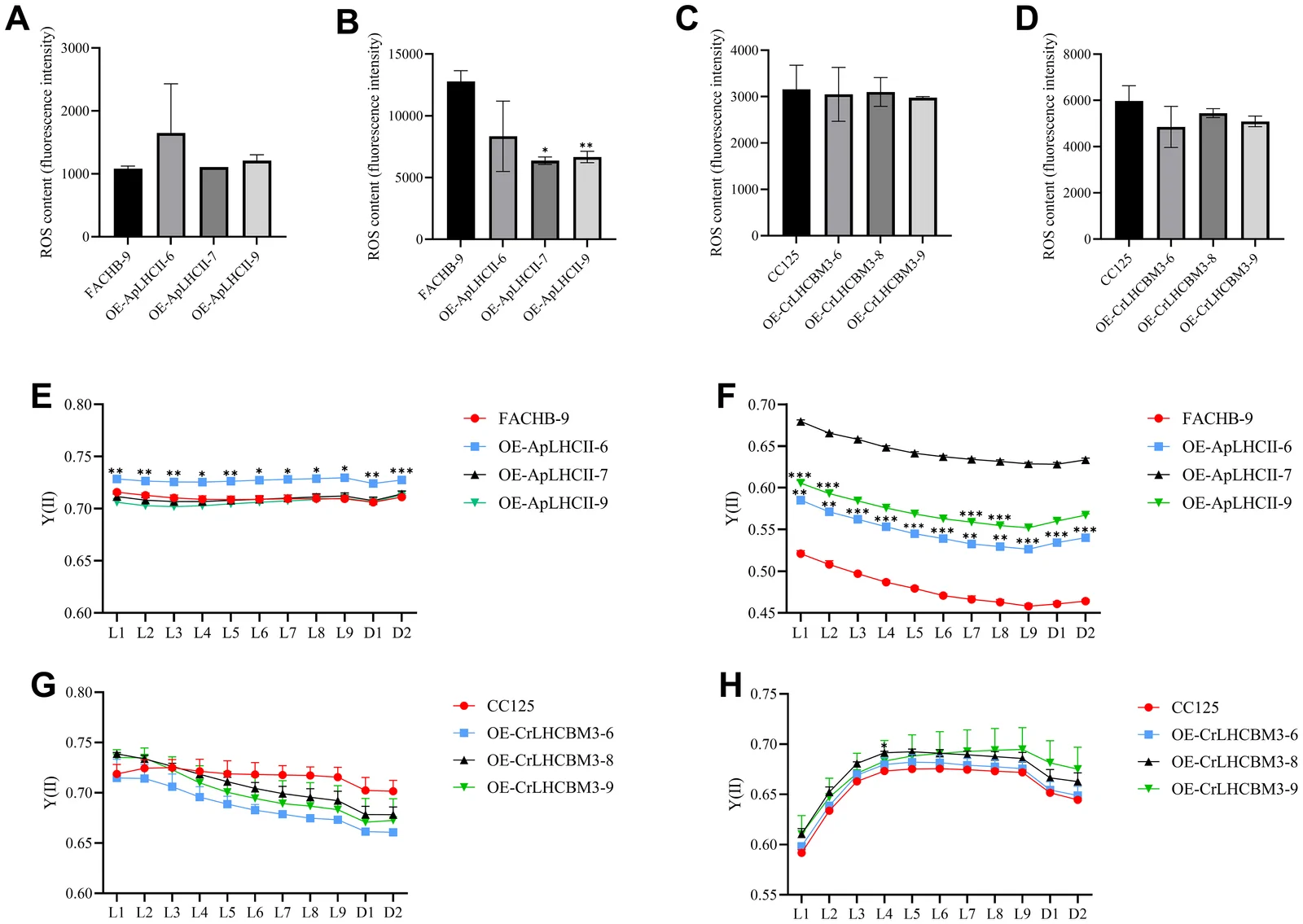
Overexpression of the major LHCII gene differentially affects ROS levels and Y(II) in the two algae under normal and ammonium stress conditions: significant effects in **(A)** pyrenoidosa (except for a few individual lines), while effects in *C. reinhardtii* did not reach significance. **(A)** ROS content of *A. pyrenoidosa* under normal conditions; **(B)** ROS content of *A. pyrenoidosa* under ammonium stress; **(C)** ROS content of *C. reinhardtii* under normal conditions; **(D)** ROS content of *C. reinhardtii* under ammonium stress; Differences between each transgenic line and WT were assessed by Welch’s ANOVA with Dunnett’s T3 *post-hoc* test. *P < 0.05, **P < 0.01, ***P < 0.001 vs respective WT; ns, not significant. **(E)** Y(II) changes in *A. pyrenoidosa* during light acclimation (L1–L9) and dark recovery (D1–D2) under normal conditions; **(F)** Y(II) changes in *A. pyrenoidosa* under ammonium stress; **(G)** Y(II) changes in *C. reinhardtii* under normal conditions; **(H)** Y(II) changes in *C. reinhardtii* under ammonium stress. Data are mean ± SD (*n* = 3). Strain and interaction effects were assessed by two-way ANOVA (Time × Strain) followed by Dunnett’s *post-hoc* test.

SOD activity assay (**Supplementary Figure A4**) revealed an alga-specific regulatory pattern of overexpression of the major LHCII gene on SOD activity under the tested conditions: mainly up-regulated in *A. pyrenoidosa* and down-regulated in *C. reinhardtii*. In *A. pyrenoidosa*, under normal conditions, only line OE-ApLHCII-7 showed significantly higher SOD activity than the wild-type; under ammonium stress, except for line OE-ApLHCII-7 which showed significantly lower SOD activity, the other OE lines showed significantly higher SOD activity. In *C. reinhardtii*, SOD activity in OE lines was significantly lower than that in the wild-type under both normal and ammonium stress conditions.

Notably, the changes in SOD activity were not negatively correlated with ROS levels in either alga under ammonium stress. In *A. pyrenoidosa*, several OE lines showed significantly increased SOD activity while ROS was reduced; however, reduced ROS was also observed in lines with unchanged or decreased SOD activity. In *C. reinhardtii*, all OE lines showed significantly lower SOD activity yet ROS levels also trended lower.

Changes in PSII effective photochemical quantum yield [Y(II)] (**Figures 6E–H**) showed that for *A. pyrenoidosa* under normal conditions, line OE-ApLHCII-6 had slightly higher Y(II) at all time points than the wild-type, while other OE lines showed little difference. Under ammonium stress, all OE lines exhibited consistently higher Y(II) than the wild-type. For *C. reinhardtii* under normal conditions, OE lines generally showed slightly lower Y(II) than the wild-type; under ammonium stress, all OE lines had higher Y(II) than the wild-type, but the differences were not significant.

Under ammonium stress, Fv/Fm was significantly higher in *ApLHCII* overexpression lines than in the wild type in *A. pyrenoidosa*, whereas no significant difference was observed under normal conditions. In *C. reinhardtii*, Fv/Fm did not differ significantly between CrLHCBM3 overexpression lines and the wild type under either condition (**Figure 7**).

**Figure 7:**
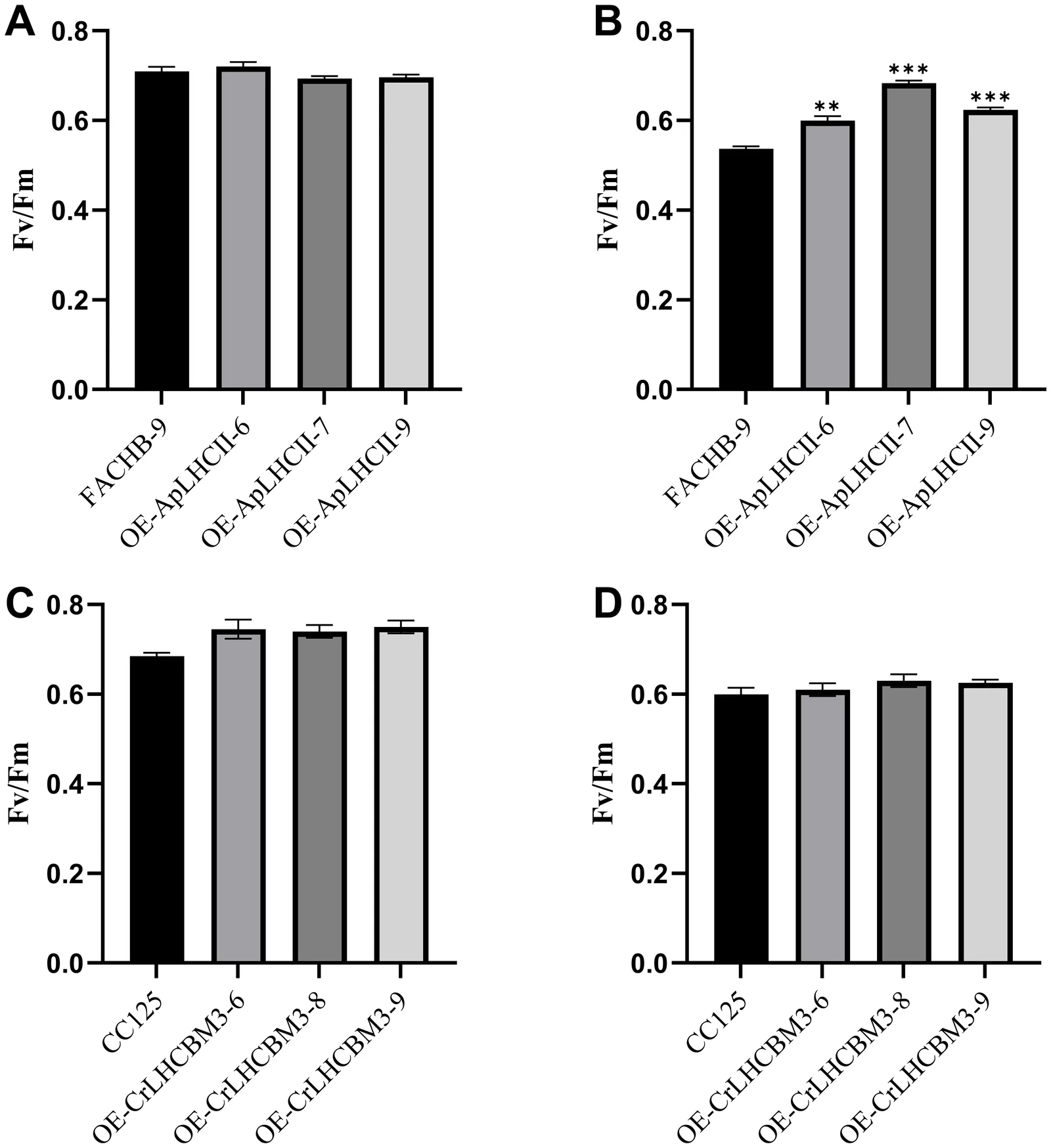
*ApLHCII* overexpression increases Fv/Fm in *A. pyrenoidosa* under ammonium stress, with no significant effect in *C. reinhardtii*. **(A)** *A. pyrenoidosa* under normal condition; **(B)** *A. pyrenoidosa* under ammonium stress (17.6 mM NH_4_Cl); **(C)** *C. reinhardtii* under normal condition; **(D)** *C. reinhardtii* under ammonium stress (35 mM NH_4_Cl). Fv/Fm was measured after 15 min of dark adaptation. Data are mean ± SD (n = 3). Differences between each OE line and WT were assessed by Welch’s ANOVA with Dunnett’s T3 *post-hoc* test. *P < 0.05, **P < 0.01, ***P < 0.001, vs WT; ns, not significant.

## 4 Discussion

Comparison of transcriptomic responses in *A. pyrenoidosa*, *C. reinhardtii* and rice showed that the photosynthesis-antenna protein pathway was detected in all three species and significantly enriched in *C. reinhardtii* and rice under ammonium stress (Alencar et al., 2019), suggesting that disturbance of the light-harvesting system represents a conserved response module from microalgae to higher plants, consistent with the role of PSII antenna regulation as a universal stress acclimation mechanism in higher plants (Borisova-Mubarakshina et al., 2020; Vetoshkina et al., 2023). Within this pathway, cross-species ortholog comparison identified eight candidate genes forming three ortholog groups within two cross-species orthogroups (**Tables 1**, **2**). As described in Results, the major LHCII group was prioritized for functional validation based on its mechanistic relevance to PSII damage and its strongest cross-species downregulation. In rice, the repression was particularly drastic: *LHCB2* and *LHCA4* transcription was essentially completely suppressed (0/1/1 raw counts in the three stress replicates; **Table 2**). This near-complete shutdown of the photosynthetic antenna system is consistent with the known inhibition of PSII electron transport and damage to the oxygen-evolving complex under ammonium toxicity (Wang et al., 2019; Xie et al., 2025), and with the general acclimation strategy of reducing light harvesting to limit photo-oxidative damage. Notably, only ten genes highly expressed in the control group showed such complete silencing, and eight of these ten were photosynthesis-related, indicating that the repression was pathway-specific rather than global (**Supplementary Table 3**). In *C. reinhardtii*, *LHCBM3* showed a downregulation trend (log_2_FC = −1.26, padj = 0.171; **Table 2**) and was retained as the representative of the major LHCII group on orthology and functional grounds, as its orthologs were significantly downregulated in both *A. pyrenoidosa* and rice. The LHCA4 groups, although also downregulated, were not taken forward for functional validation, because LHCA4 belongs to the PSI antenna and is less directly linked to the ammonium toxicity phenotypes. Phylogenetic analysis revealed that these genes belong to different LHC subfamilies in different species — *LHCBM3* in *C. reinhardtii*, *LHCB2* in rice, and a major LHCII member in *A. pyrenoidosa* — indicating that ammonium stress downregulates the LHCII antenna apparatus broadly, rather than a specific subfamily member. Because this gene encodes a major PSII light-harvesting antenna protein involved in chlorophyll binding, light capture and photoprotection, its conserved transcriptional response, which parallels the coordinated downregulation of LHCII antenna genes observed in *C. reinhardtii* under nitrogen deprivation (Juergens et al., 2015), made it a strong candidate for functional analysis.

Functional characterization of the major LHCII gene overexpression lines showed that the transgene positively affected growth-related traits in both algae under normal conditions, consistent with reports in higher plants that *LHCB1* promotes photosynthesis (Wang et al., 2025), and with the demonstrated feasibility of enhancing abiotic stress tolerance in *C. reinhardtii* through transgenic overexpression (Yazdani et al., 2021). Under ammonium stress, however, the beneficial effects were expressed differently in the two species: *A. pyrenoidosa* mainly showed increased cell density and chlorophyll accumulation, whereas *C. reinhardtii* showed a stronger relative increase in fresh weight together with improved chlorophyll content. These results indicate that although the major LHCII gene contributes positively to ammonium tolerance in both algae, the physiological traits most strongly influenced by the transgene differ between species, although the possibility cannot be excluded that the milder effects observed in *C. reinhardtii* partly reflect the specific ammonium concentration tested for this alga.

A notable feature of this study is that the major LHCII gene was transcriptionally downregulated under ammonium stress, whereas its overexpression improved performance under ammonium stress. This apparent contrast suggests that stress-induced downregulation of the major LHCII gene may reflect either a protective adjustment of the light-harvesting system to prevent excess light absorption under metabolic stress (Borisova-Mubarakshina et al., 2020)or a consequence of photosynthetic impairment under ammonium toxicity. It should be noted that LHCII downregulation is also a well-established response to nitrogen deprivation in *C. reinhardtii*. In our experiments, however, nitrogen availability was not reduced by the ammonium treatments: both control and stress conditions of *C. reinhardtii* used TAP medium containing ammonium as the nitrogen source (7.5 mM in the control; an additional 35 mM NH_4_Cl in the stress condition); in *A. pyrenoidosa*, nitrate (17.65 mM NaNO_3_ in BG11) was replaced by an equimolar concentration of NH_4_Cl (17.6 mM); and the rice ammonium stress group received additional NH_4_Cl (4 mM) relative to the control. The observed LHCII downregulation therefore represents a response to ammonium excess rather than a general nitrogen-deprivation response. In either case, the improved performance of overexpression lines supports the idea that maintaining a higher expression level of the major LHCII gene can partly buffer the photosynthetic system against ammonium stress.

Physiological analyses indicated that the beneficial effects of LHCII overexpression under ammonium stress were associated with changes in photochemical performance and oxidative stress responses, but the downstream physiological manifestations were species-dependent under the tested conditions. In *A. pyrenoidosa*, LHCII overexpression was associated with lower ROS accumulation in most lines, consistently higher Y(II), and significantly elevated Fv/Fm under ammonium stress(**Figure 7**). In *C. reinhardtii*, by contrast, the trends in ROS reduction and Y(II) improvement were weaker and largely non-significant, Fv/Fm did not differ between overexpression lines and the wild type, and the reduction in ROS was not accompanied by consistent upregulation of SOD activity. These observations are consistent with the distinct functional roles of LHCBM isoforms in thermal dissipation and state transitions in this species (Elrad et al., 2002; Ferrante et al., 2012). Specifically, in *A. pyrenoidosa*, the protective effect extended beyond the antenna to the PSII reaction center, whereas in *C. reinhardtii*, protection was largely confined to the antenna level. Therefore, our data support a model in which LHCII genes represent a shared component of the ammonium stress transcriptional response, whereas the downstream physiological manifestations of overexpression differ between microalgal species.

Cross-species transcriptomic comparison identified a shared transcriptional response of major LHCII-related genes to ammonium stress. Overexpression of the corresponding major LHCII genes improved growth- and chlorophyll-related traits in two microalgae, although the physiological responses differed under the tested culture conditions. These findings provide a candidate gene for engineering ammonium-tolerant microalgae and a conceptual framework for understanding conserved and divergent components of ammonium stress responses under the tested conditions.

This study examined only three photosynthetic species, and whether the findings extend to other microalgae and higher plants requires direct validation in additional species. Nevertheless, several lines of evidence suggest that the identified response may have broader relevance. First, the LHCII antenna system is evolutionarily ancient and conserved across green algae and land plants. Second, downregulation of LHCB genes has been documented under various abiotic stresses in multiple algal and plant systems, indicating that antenna remodeling is a general stress response rather than a species-specific peculiarity. Third, the two algae used here belong to distinct chlorophyte lineages (Trebouxiophyceae and Chlorophyceae), and their convergent responses, together with those of the higher plant reference, suggest that the mechanism is unlikely to be restricted to these species. In addition, transgene insertion sites were not determined in this study; however, the consistent beneficial phenotypes across multiple independent transgenic lines (10 for *A. pyrenoidosa* and 6 for *C. reinhardtii*) verified by PCR and qRT-PCR suggest that the observed effects were not attributable to a single transformation event.

This study has several limitations. First, the three species were examined under different experimental conditions — *A. pyrenoidosa* and *C. reinhardtii* were grown in different media (BG11 vs. TAP) with different ammonium concentrations (17.6 vs. 35 mM), and the rice data originated from a different experimental system — so the species-associated patterns observed here may partly reflect these condition differences, and cross-species differences should not be interpreted as intrinsic species properties. Second, empty-vector control lines were not generated, so effects of the transformation process itself cannot be formally excluded; however, the consistent phenotypes across multiple independent transgenic lines (10 for *A. pyrenoidosa*, 6 for *C. reinhardtii*) with distinct insertion sites reduce the likelihood of such artifacts. Third, overexpression was verified at the transcript level by qRT-PCR; protein-level confirmation was not performed because no commercial antibody specific to *LHCBM3* is available, although the observed phenotypic changes are consistent with functional antenna protein accumulation. Nevertheless, the LHCII antenna gene downregulation observed across evolutionarily distant species in independent experimental systems is consistent with the view that disturbance of the light-harvesting system represents a conserved response module from microalgae to higher plants.

## Data Availability

The datasets presented in this study can be found in online repositories. The names of the repository/repositories and accession number(s) can be found below: NGDC Genome Sequence Archive: CRA045709 (https://ngdc.cncb.ac.cn/gsa/search?searchTerm=CRA045709), CRA045685 (https://ngdc.cncb.ac.cn/gsa/search?searchTerm=CRA045685).
