# Supplementary material for: Cross-species transcriptomics and transgenic analyses implicate major LHCII genes in microalgal responses to ammonium stress
Source: Front Plant Sci. 2026 Sep 24;17:1952007. doi: 10.3389/fpls.2026.1952007 (PMC13648037; doi:10.3389/fpls.2026.1952007)
Supplement: Supplementary file 1 [file DataSheet1.pdf]

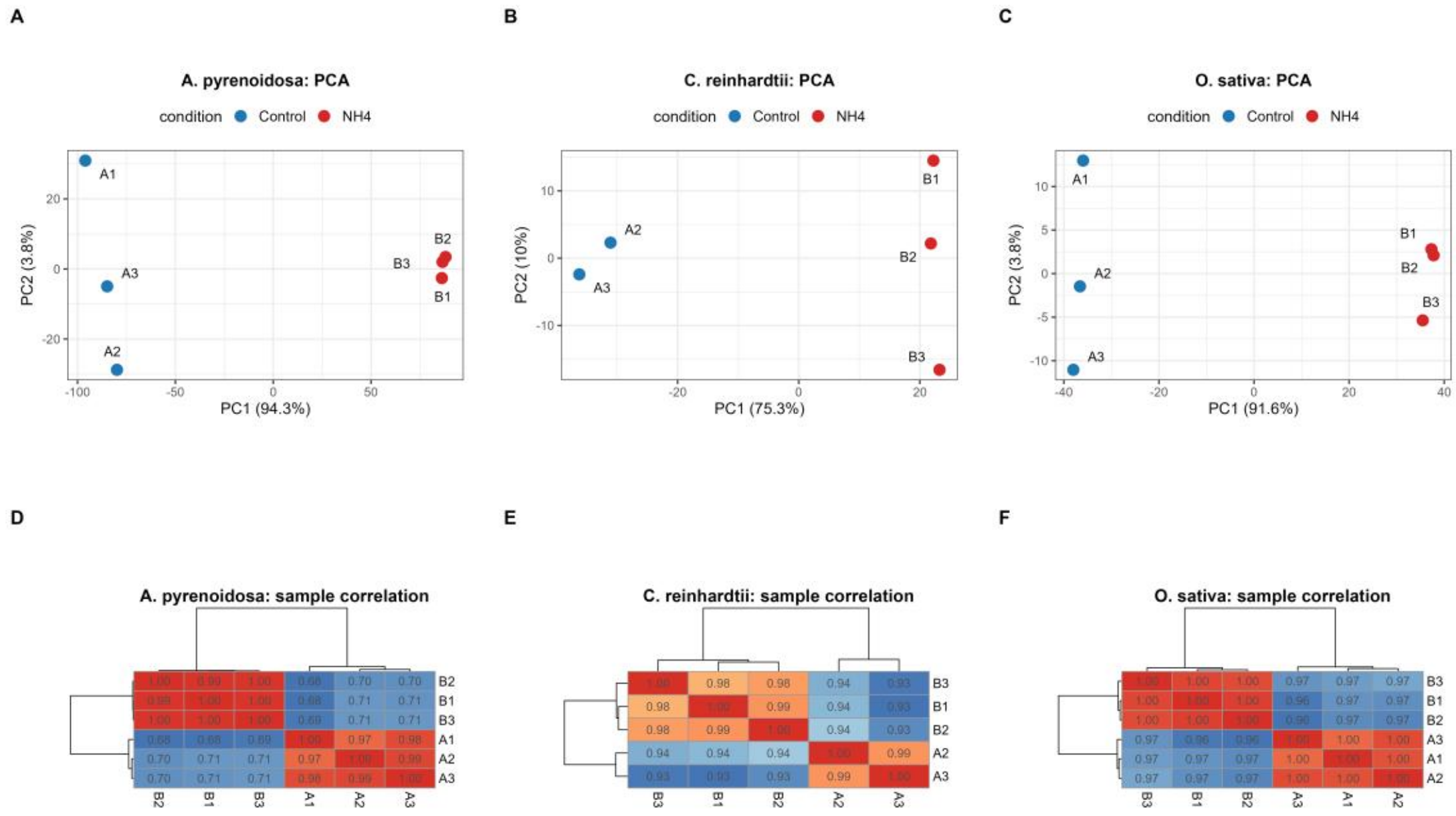

Figure A1 Quality control of RNA-seq biological replicates. (A–C) Principal component analysis (PCA) of *A. pyrenoidosa*, *C. reinhardtii*, and *O. sativa* samples, respectively. (D–F) Pearson correlation heatmaps of biological replicates for the three species. Biological replicates clustered by treatment group in PCA. Within-group Pearson correlations ranged from 0.97 to 1.00 for *A. pyrenoidosa*, 0.98 to 0.99 for *C. reinhardtii*, and 0.99 to 1.00 for *O. sativa*. For *C. reinhardtii*, the analyses were based on the two control and three ammonium-stress replicates (the single-end A1 replicate was not used).
