# Supplementary material for: Cross-species transcriptomics and transgenic analyses implicate major LHCII genes in microalgal responses to ammonium stress
Source: Front Plant Sci. 2026 Sep 24;17:1952007. doi: 10.3389/fpls.2026.1952007 (PMC13648037; doi:10.3389/fpls.2026.1952007)
Supplement: Supplementary file 2 [file DataSheet2.pdf]

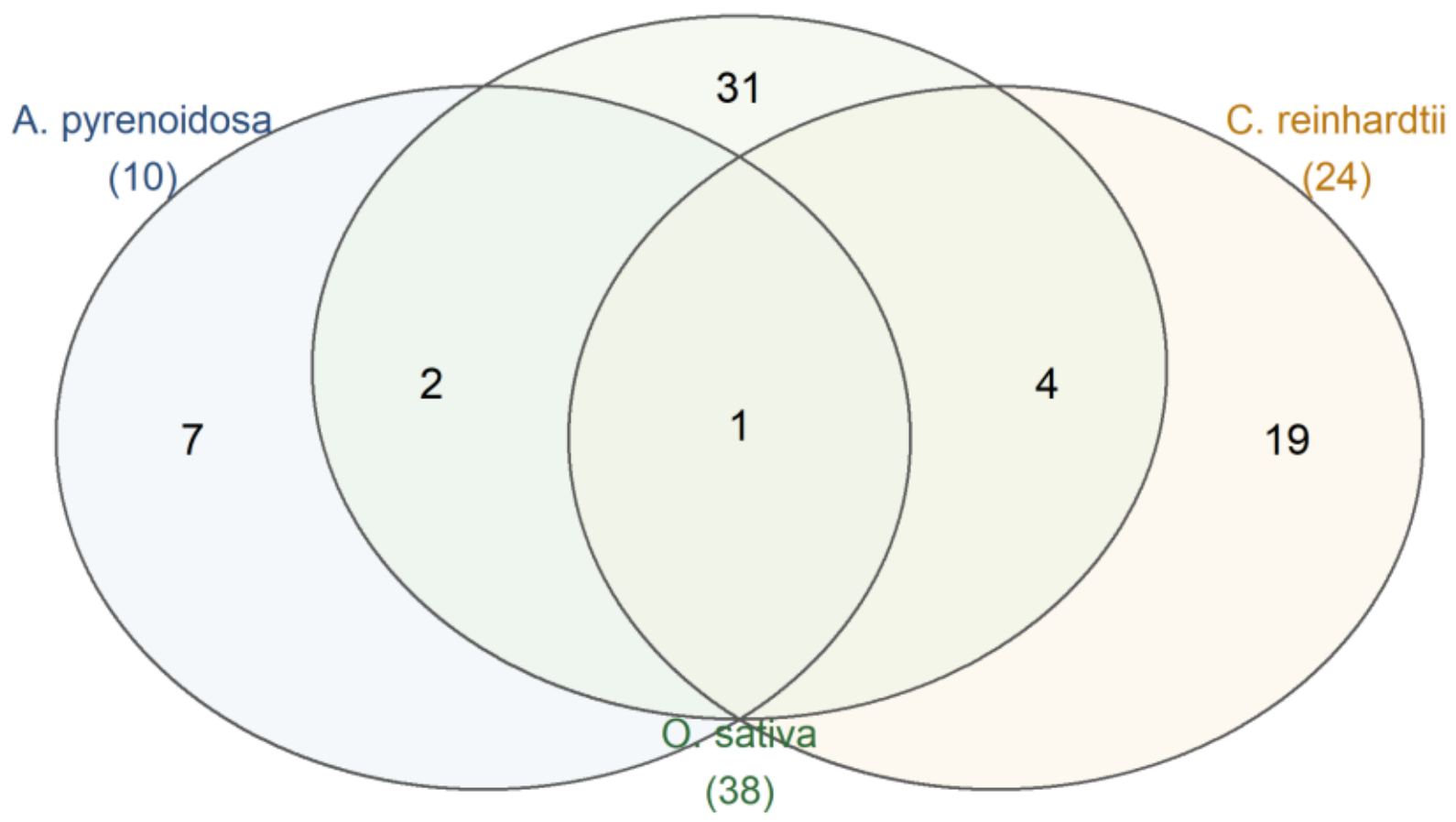

Figure A2 Overlap of significantly enriched KEGG pathways (P < 0.05) among the three species. Numbers of significantly enriched pathways: *A. pyrenoidosa*, 10; *C. reinhardtii*, 24; *O. sativa*, 38. Starch and sucrose metabolism (ko00500) was the only pathway significantly enriched in all three species.
