# Supplementary material for: Cross-species transcriptomics and transgenic analyses implicate major LHCII genes in microalgal responses to ammonium stress
Source: Front Plant Sci. 2026 Sep 24;17:1952007. doi: 10.3389/fpls.2026.1952007 (PMC13648037; doi:10.3389/fpls.2026.1952007)
Supplement: Supplementary file 3 [file DataSheet3.pdf]

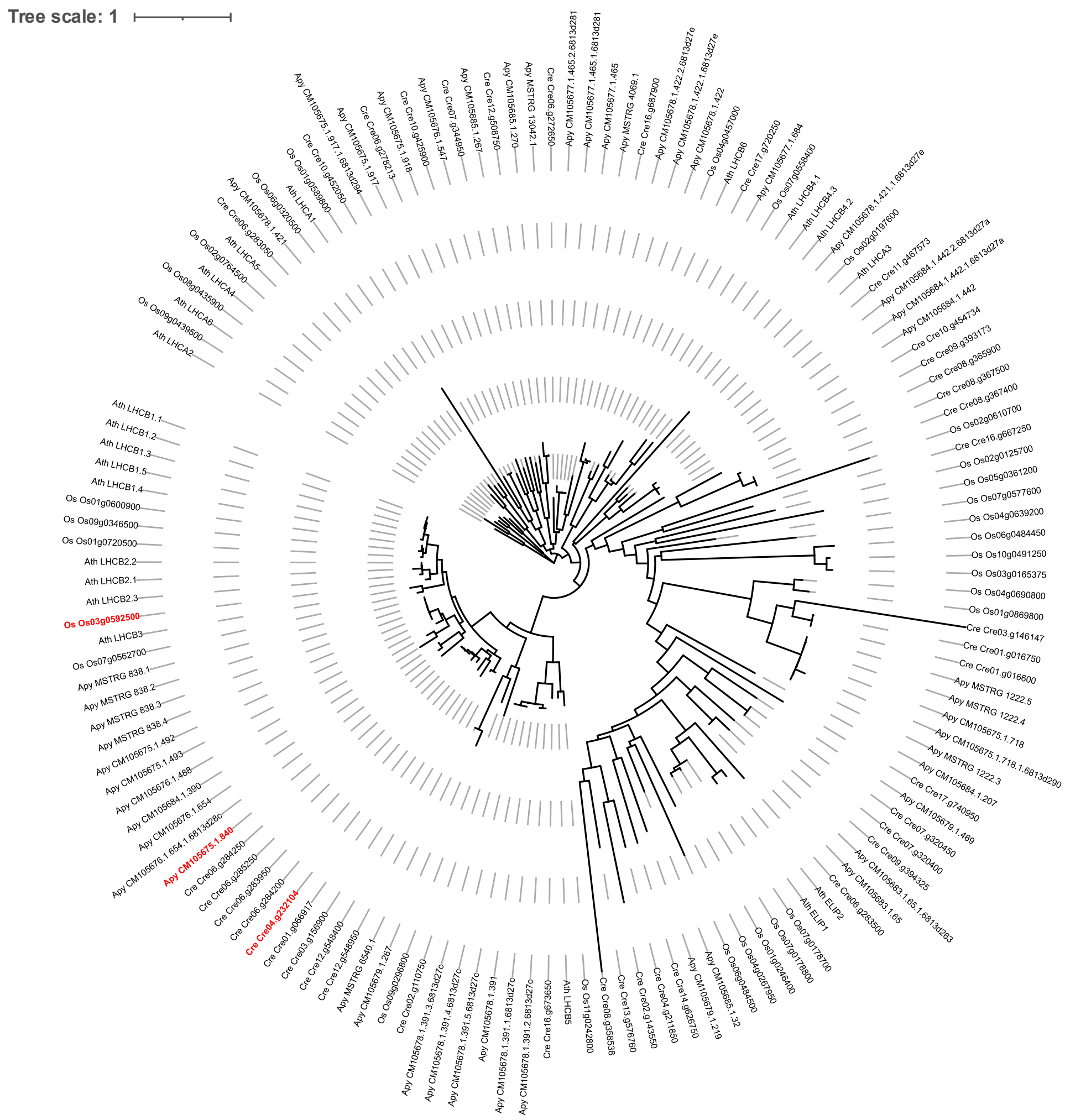

Figure A3 Phylogenetic analysis of the LHCB family across four species. Maximum-likelihood phylogenetic tree of LHCB/LHCBM and LHCA protein sequences from *Arabidopsis thaliana* (22 sequences, including LHCB1–LHCB6 and LHCA1–LHCA6 as reference subfamilies), *Auxenochlorella pyrenoidosa* (50 sequences), *Chlamydomonas reinhardtii* (40 sequences), and *Oryza sativa* (30 sequences). All sequences were identified by the presence of the Chloroa_b-bind domain (PF00504). Protein sequences were aligned using MAFFT v7 with the --auto algorithm; columns with >50% gaps were removed. The tree was constructed using FastTree v2 with the LG+Gamma substitution model. ELIP1 and ELIP2 from *A. thaliana* were used as outgroup. The three genes identified through cross-species transcriptomic comparison are indicated by red labels: *Os03g0592500* (*LHCB2*), *Cre04.g232104* (*LHCBM3*), and the *A. pyrenoidosa* gene *CM105675.1.840* (LHCII family member). SH-like local support values >0.70 are shown at nodes.
