# Supplementary material for: Cross-species transcriptomics and transgenic analyses implicate major LHCII genes in microalgal responses to ammonium stress
Source: Front Plant Sci. 2026 Sep 24;17:1952007. doi: 10.3389/fpls.2026.1952007 (PMC13648037; doi:10.3389/fpls.2026.1952007)
Supplement: Supplementary file 4 [file DataSheet4.pdf]

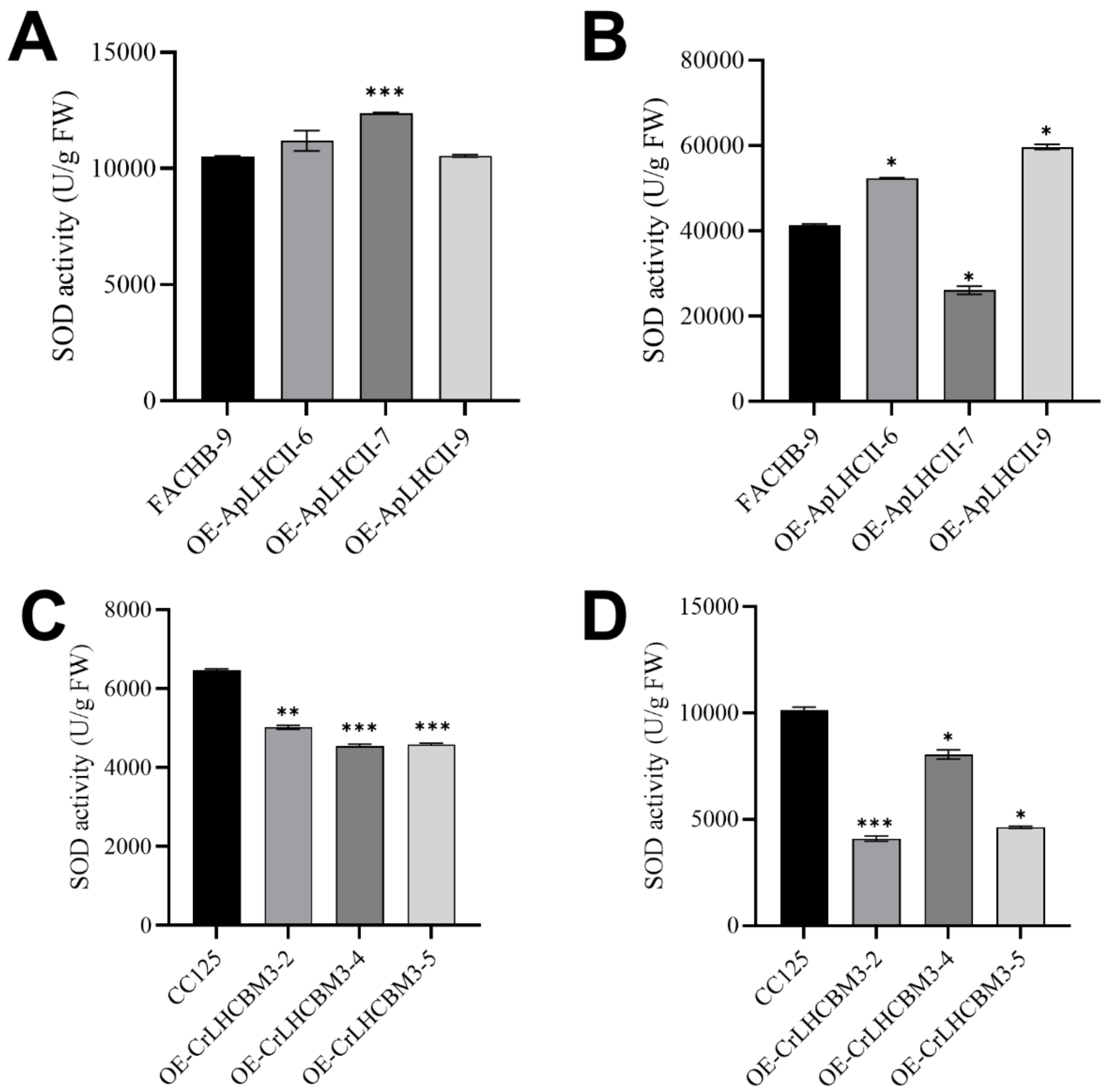

Figure A4 SOD activity in *A. pyrenoidosa* and *C. reinhardtii* under normal and ammonium stress conditions. (A) SOD activity in *A. pyrenoidosa* under normal conditions; (B) SOD activity in *A. pyrenoidosa* under ammonium stress; (C) SOD activity in *C. reinhardtii* under normal conditions; (D) SOD activity in *C. reinhardtii* under ammonium stress. Data are mean ± SD (n = 3). Differences between each transgenic line and WT were assessed by Welch's ANOVA with Dunnett's T3 post-hoc test. *$P < 0.05$, **$P < 0.01$, ***$P < 0.001$ vs respective WT; ns, not significant.
