# Supplementary material for: Cross-species transcriptomics and transgenic analyses implicate major LHCII genes in microalgal responses to ammonium stress
Source: Front Plant Sci. 2026 Sep 24;17:1952007. doi: 10.3389/fpls.2026.1952007 (PMC13648037; doi:10.3389/fpls.2026.1952007)
Supplement: Supplementary file 5 [file Table2.docx]

**Table S2** Complete list of the 42 strict DEGs annotated to the photosynthesis-antenna protein pathway and their inclusion in the candidate ortholog groups

| Gene ID | Species | Annotation | KO | log₂FC | padj | Reason |
| --- | --- | --- | --- | --- | --- | --- |
| evm.TU.CM105684.1.390 | A. pyrenoidosa | LHCB1/LHCB2 | K08912/K08913 | 4.57 | 7.17E-53 | Candidate OG member but upregulated (opposite to focal downregulation pattern) |
| evm.TU.CM105675.1.492 | A. pyrenoidosa | LHCB1/LHCB2 | K08912/K08913 | -1.12 | 0.005465478 | Broad LHCB annotation; not the defined major LHCII focal gene |
| evm.TU.CM105676.1.654 | A. pyrenoidosa | LHCB1/LHCB2 | K08912/K08913 | -1.42 | 9.53E-06 | Broad LHCB annotation; not the defined major LHCII focal gene |
| evm.TU.CM105675.1.840 | A. pyrenoidosa | LHCII | K08912/K08913 | -1.49 | 4.65E-05 | Focal subfamily (major LHCII); downregulated strict DEG |
| evm.TU.CM105676.1.488 | A. pyrenoidosa | LHCB1/LHCB2 | K08912/K08913 | -1.61 | 8.07E-07 | Broad LHCB annotation; not the defined major LHCII focal gene |
| Cre01.g066917.v5.5 | C. reinhardtii | LHCB1/LHCB2 | K08912/K08913 | -1.26 | 8.05E-05 | Broad LHCB annotation; not the defined major LHCII focal gene |
| Cre06.g285250.v5.5 | C. reinhardtii | LHCB1/LHCB2 | K08912/K08913 | -1.46 | 1.59E-07 | Broad LHCB annotation; not the defined major LHCII focal gene |
| Cre12.g548400.v5.5 | C. reinhardtii | LHCB1/LHCB2 | K08912/K08913 | -1.63 | 0.03701921 | Broad LHCB annotation; not the defined major LHCII focal gene |
| Cre12.g548950.v5.5 | C. reinhardtii | LHCB1/LHCB2 | K08912/K08913 | -1.67 | 0.028042394 | Broad LHCB annotation; not the defined major LHCII focal gene |
| Cre06.g283950.v5.5 | C. reinhardtii | LHCB1/LHCB2 | K08912/K08913 | -3.02 | 0.01193228 | Broad LHCB annotation; not the defined major LHCII focal gene |
| Cre06.g284250.v5.5 | C. reinhardtii | LHCB1/LHCB2 | K08912/K08913 | -3.25 | 0.003729607 | Broad LHCB annotation; not the defined major LHCII focal gene |
| Os01g0720500 | O. sativa | LHCB1 | K08912 | -1.79 | 5.37E-41 | Broad LHCB annotation; not the defined major LHCII focal gene |
| Os01g0600900 | O. sativa | LHCB1 | K08912 | -2.51 | 2.02E-98 | Broad LHCB annotation; not the defined major LHCII focal gene |
| Os09g0346500 | O. sativa | LHCB1 | K08912 | -6.33 | 4.36E-193 | Broad LHCB annotation; not the defined major LHCII focal gene |
| Os07g0562700 | O. sativa | LHCB3 | K08914 | -6.76 | 2.30E-60 | Broad LHCB annotation; not the defined major LHCII focal gene |
| Os03g0592500 | O. sativa | LHCB2 | K08913 | -9.74 | 3.01E-20 | Focal subfamily (major LHCII); downregulated strict DEG |
| evm.TU.CM105677.1.465 | A. pyrenoidosa | LHCA4/LHCA5 | K08910/K08911 | -1.03 | 0.007652876 | LHCA subfamily with dual/other KO; outside focal single-K08910 scope |
| evm.TU.CM105676.1.547 | A. pyrenoidosa | LHCA4 | K08910 | -1.26 | 0.006091301 | Focal subfamily (LHCA4, single K08910); downregulated strict DEG |
| evm.TU.CM105675.1.918 | A. pyrenoidosa | LHCA4 | K08910 | -1.28 | 8.32E-05 | Focal subfamily (LHCA4, single K08910); downregulated strict DEG |
| Cre06.g278213.v5.5 | C. reinhardtii | LHCA4 | K08910 | -1.4 | 0.044273886 | Focal subfamily (LHCA4, single K08910); downregulated strict DEG |
| Cre10.g425900.v5.5 | C. reinhardtii | LHCA4 | K08910 | -1.41 | 2.16E-08 | Focal subfamily (LHCA4, single K08910); downregulated strict DEG |
| Cre06.g272650.v5.5 | C. reinhardtii | LHCA4/LHCA5 | K08910/K08911 | -1.51 | 6.03E-09 | LHCA subfamily with dual/other KO; outside focal single-K08910 scope |
| Cre16.g687900.v5.5 | C. reinhardtii | LHCA5 | K08911 | -1.58 | 6.81E-07 | Minor antenna subfamily (LHCA5); outside the focal candidate scope |
| Cre10.g452050.v5.5 | C. reinhardtii | LHCA2/LHCA4 | K08908/K08910 | -1.7 | 3.28E-10 | LHCA subfamily with dual/other KO; outside focal single-K08910 scope |
| Os07g0577600 | O. sativa | LHCA2 | K08908 | -4.62 | 6.56E-51 | LHCA subfamily with other KO (LHCA2); outside focal single-K08910 scope |
| Os08g0435900 | O. sativa | LHCA4 | K08910 | -8.97 | 3.42E-17 | Focal subfamily (LHCA4, single K08910); downregulated strict DEG |
| evm.TU.CM105679.1.267 | A. pyrenoidosa | LHCQ | K14172 | 1.67 | 2.11E-07 | Upregulated; direction opposite to the candidate downregulation pattern |
| evm.TU.CM105685.1.270 | A. pyrenoidosa | LHCA1 | K08907 | -1.2 | 3.78E-05 | Non-focal PSI antenna subfamily (LHCA1) |
| evm.TU.CM105678.1.391 | A. pyrenoidosa | LHCB5(CP26) | K08916 | -1.35 | 0.000978414 | Minor PSII antenna protein (CP29/CP26/CP24); outside the focal candidate scope |
| evm.TU.CM105677.1.684 | A. pyrenoidosa | LHCB4(CP29) | K08915 | -1.37 | 0.000896902 | Minor PSII antenna protein (CP29/CP26/CP24); outside the focal candidate scope |
| evm.TU.CM105684.1.442 | A. pyrenoidosa | LHCA3 | K08909 | -1.52 | 1.29E-05 | Non-focal PSI antenna subfamily (LHCA3) |
| evm.TU.CM105678.1.421 | A. pyrenoidosa | LHCA1 | K08907 | -1.59 | 7.70E-06 | Non-focal PSI antenna subfamily (LHCA1) |
| Cre10.g454734.v5.5 | C. reinhardtii | LHCA3 | K08909 | 1.38 | 1.19E-09 | Upregulated; direction opposite to the candidate downregulation pattern |
| Cre12.g508750.v5.5 | C. reinhardtii | LHCA1 | K08907 | -1.4 | 1.77E-05 | Non-focal PSI antenna subfamily (LHCA1) |
| Cre07.g344950.v5.5 | C. reinhardtii | LHCA5 | K08911 | -1.5 | 1.90E-07 | Minor antenna subfamily (LHCA5); outside the focal candidate scope |
| Cre06.g283050.v5.5 | C. reinhardtii | LHCA1 | K08907 | -1.89 | 5.11E-12 | Non-focal PSI antenna subfamily (LHCA1) |
| Cre11.g467573.v5.5 | C. reinhardtii | LHCA3 | K08909 | -2 | 4.49E-14 | Non-focal PSI antenna subfamily (LHCA3) |
| Os11g0242800 | O. sativa | LHCB5(CP26) | K08916 | -1.6 | 3.63E-41 | Minor PSII antenna protein (CP29/CP26/CP24); outside the focal candidate scope |
| Os04g0457000 | O. sativa | LHCB6(CP24) | K08917 | -5.04 | 1.44E-37 | Minor PSII antenna protein (CP29/CP26/CP24); outside the focal candidate scope |
| Os07g0558400 | O. sativa | LHCB4(CP29) | K08915 | -6.35 | 4.66E-48 | Minor PSII antenna protein (CP29/CP26/CP24); outside the focal candidate scope |
| Os06g0320500 | O. sativa | LHCA1 | K08907 | -8.86 | 2.03E-32 | Non-focal PSI antenna subfamily (LHCA1) |
| Os02g0197600 | O. sativa | LHCA3 | K08909 | -10.45 | 1.58E-17 | Non-focal PSI antenna subfamily (LHCA3) |

Strict DEGs were defined as |log₂FC| > 1 and padj < 0.05. The seven genes marked in red are members of the two focal antenna orthogroups (major LHCII and LHCA4) carried forward to Table 1. The remaining genes are reported for completeness and corroborate the broad antenna response to ammonium stress. KEGG KO identifiers: K08907, LHCA1; K08908, LHCA2; K08909, LHCA3; K08910, LHCA4; K08911, LHCA5; K08912/K08913, LHCB1/2. NA, no KO assignment.
