# Supplementary material for: Cross-species transcriptomics and transgenic analyses implicate major LHCII genes in microalgal responses to ammonium stress
Source: Front Plant Sci. 2026 Sep 24;17:1952007. doi: 10.3389/fpls.2026.1952007 (PMC13648037; doi:10.3389/fpls.2026.1952007)
Supplement: Supplementary file 6 [file Table3.docx]

**Table S3** The ten genes highly expressed in the control group that were completely silenced under ammonium stress in rice

| Gene ID | Annotation | A1 | A2 | A3 | B1 | B2 | B3 |
| --- | --- | --- | --- | --- | --- | --- | --- |
| *OS03G0592500* | PSII type II chlorophyll a/b binding protein（*LHCB2*） | 723 | 535 | 499 | 0 | 1 | 1 |
| *OS12G0291100* | Rubisco small subunit 3 | 616 | 391 | 450 | 0 | 1 | 0 |
| *OS08G0435900* | LHCI subunit（*LHCA4*） | 428 | 278 | 325 | 0 | 1 | 1 |
| *OS12G0420400* | PSI reaction centre subunit XI | 388 | 262 | 320 | 1 | 0 | 0 |
| *OS08G0200300* | PSII 10 kDa polypeptide | 351 | 234 | 284 | 1 | 0 | 0 |
| *OS02G0197600* | Chlorophyll a/b-binding protein type III | 341 | 227 | 227 | 0 | 0 | 0 |
| *OS07G0148900* | Photosystem I protein-like | 294 | 204 | 227 | 0 | 0 | 0 |
| *OS04G0481700* | MDR-like ABC transporter | 137 | 161 | 135 | 0 | 0 | 0 |
| *OS04G0308500* | H0211A12.12 protein | 0 | 186 | 232 | 0 | 0 | 0 |
| *OS12G0189400* | PSI reaction centre subunit N | 164 | 84 | 95 | 0 | 0 | 0 |

Note: Counts are raw mapped reads per gene. Genes shown were highly expressed in the control group (mean count > 100) but completely silenced under ammonium stress (≤1 read in all three stress replicates). Eight of the ten genes are photosynthesis-related, including three light-harvesting antenna genes (*Os03g0592500*, *Os08g0435900*, *Os02g0197600*).
