# Supplementary material for: Cross-species transcriptomics and transgenic analyses implicate major LHCII genes in microalgal responses to ammonium stress
Source: Front Plant Sci. 2026 Sep 24;17:1952007. doi: 10.3389/fpls.2026.1952007 (PMC13648037; doi:10.3389/fpls.2026.1952007)
Supplement: Supplementary file 7 [file Table4.docx]

**Table S4** Whole-day comparisons of OD₆₈₀ between LHCII overexpression lines and WT in *A. pyrenoidosa* under normal culture conditions

| Day | WT mean | OE-ApLHCII-1 | OE-ApLHCII-3 | OE-ApLHCII-4 | OE-ApLHCII-6 | OE-ApLHCII-7 | OE-ApLHCII-8 | OE-ApLHCII-9 | OE-ApLHCII-10 | OE-ApLHCII-13 | OE-ApLHCII-15 |
| --- | --- | --- | --- | --- | --- | --- | --- | --- | --- | --- | --- |
| 1 | 0.052 | >0.999^ns^ | >0.999^ns^ | >0.999^ns^ | >0.999^ns^ | >0.999^ns^ | >0.999^ns^ | >0.999^ns^ | >0.999^ns^ | >0.999^ns^ | >0.999^ns^ |
| 2 | 0.072 | >0.999^ns^ | >0.999^ns^ | >0.999^ns^ | >0.999^ns^ | >0.999^ns^ | >0.999^ns^ | >0.999^ns^ | >0.999^ns^ | >0.999^ns^ | >0.999^ns^ |
| 3 | 0.117 | 0.16^ns^ | 0.997^ns^ | 0.917^ns^ | 0.773^ns^ | >0.991^ns^ | >0.942^ns^ | >0.979^ns^ | >0.981^ns^ | >0.354^ns^ | >0.993^ns^ |
| 4 | 0.174 | 0.046^*^ | 0.991^ns^ | 0.292^ns^ | 0.309^ns^ | >0.991^ns^ | 0.581^ns^ | 0.646^ns^ | 0.82^ns^ | 0.006^**^ | 0.82^ns^ |
| 5 | 0.235 | <0.001^***^ | 0.048^*^ | 0.011^*^ | <0.001^***^ | 0.024^*^ | <0.001^***^ | <0.001^***^ | 0.001^**^ | <0.001^***^ | <0.001^***^ |
| 6 | 0.601 | <0.001^***^ | 0.098^ns^ | 0.113^ns^ | <0.001^***^ | <0.001^***^ | <0.001^***^ | 0.002^**^ | 0.001^**^ | <0.001^***^ | <0.001^***^ |
| 7 | 0.781 | <0.001^***^ | 0.027^*^ | 0.049^*^ | <0.001^***^ | <0.001^***^ | <0.001^***^ | <0.001^***^ | <0.001^***^ | <0.001^***^ | <0.001^***^ |
| 8 | 1.011 | <0.001^***^ | 0.123^ns^ | <0.001^***^ | <0.001^***^ | <0.001^***^ | <0.001^***^ | <0.001^***^ | 0.007^**^ | <0.001^***^ | <0.001^***^ |
| 9 | 1.383 | <0.001^***^ | 0.012^*^ | 0.019^*^ | <0.001^***^ | 0.002^**^ | <0.001^***^ | 0.403^ns^ | 0.345^ns^ | <0.001^***^ | 0.002^**^ |
| 10 | 1.503 | <0.001^***^ | <0.001^***^ | <0.001^***^ | <0.001^***^ | <0.001^***^ | <0.001^***^ | 0.007^**^ | <0.001^***^ | <0.001^***^ | <0.001^***^ |

*P* values were derived from two-way ANOVA (Time × Strain) with Dunnett's post-hoc test. n = 3 biological replicates per group. ****P* < 0.001, ***P* < 0.01, **P* < 0.05. ns, not significant (*P* ≥ 0.05).
