# Supplementary material for: Cross-species transcriptomics and transgenic analyses implicate major LHCII genes in microalgal responses to ammonium stress
Source: Front Plant Sci. 2026 Sep 24;17:1952007. doi: 10.3389/fpls.2026.1952007 (PMC13648037; doi:10.3389/fpls.2026.1952007)
Supplement: Supplementary file 8 [file Table5.docx]

**Table S5** Whole-day comparisons of OD₆₈₀ between LHCII overexpression lines and WT in *C. reinhardtii* under normal culture conditions

| Day | WT mean | OE-CrLHCBM3-1 | OE-CrLHCBM3-2 | OE-CrLHCBM3-3 | OE-CrLHCBM3-6 | OE-CrLHCBM3-8 | OE-CrLHCBM3-9 |
| --- | --- | --- | --- | --- | --- | --- | --- |
| 1 | 0.118 | >0.999^ns^ | >0.999^ns^ | >0.999^ns^ | >0.999^ns^ | >0.999^ns^ | >0.999^ns^ |
| 2 | 0.586 | 0.044^*^ | 0.184^ns^ | 0.196^ns^ | 0.265^ns^ | 0.029* | 0.381^ns^ |
| 3 | 0.917 | 0.703^ns^ | 0.009^**^ | 0.083^ns^ | 0.465^ns^ | 0.608^ns^ | 0.055^ns^ |
| 4 | 0.970 | 0.794^ns^ | <0.001^***^ | 0.207^ns^ | 0.138^ns^ | 0.006^**^ | <0.001^***^ |
| 5 | 1.072 | 0.009^**^ | 0.356^ns^ | <0.001^***^ | 0.116^ns^ | 0.807^ns^ | 0.203^ns^ |
| 6 | 1.056 | 0.003^**^ | <0.001^***^ | <0.001^***^ | 0.188^ns^ | <0.001^***^ | <0.001^***^ |
| 7 | 1.073 | <0.001^***^ | <0.001^***^ | <0.001^***^ | <0.001^***^ | <0.001^***^ | <0.001^***^ |

*P* values were derived from two-way ANOVA (Time × Strain) with Dunnett's post-hoc test. n = 3 biological replicates per group. ****P* < 0.001, ***P* < 0.01, **P* < 0.05. ns, not significant (*P* ≥ 0.05).
